# Chrna2-Martinotti Cells Synchronize Layer 5 Type A Pyramidal Cells via Rebound Excitation

**DOI:** 10.1371/journal.pbio.2001392

**Published:** 2017-02-09

**Authors:** Markus M. Hilscher, Richardson N. Leão, Steven J. Edwards, Katarina E. Leão, Klas Kullander

**Affiliations:** 1 Unit of Developmental Genetics, Department of Neuroscience, Uppsala University, Uppsala, Sweden; 2 Brain Institute, Federal University of Rio Grande do Norte, Natal, Rio Grande do Norte, Brazil; ICM - Institut du Cerveau et de la Moelle épinière, France

## Abstract

Martinotti cells are the most prominent distal dendrite–targeting interneurons in the cortex, but their role in controlling pyramidal cell (PC) activity is largely unknown. Here, we show that the nicotinic acetylcholine receptor α2 subunit (Chrna2) specifically marks layer 5 (L5) Martinotti cells projecting to layer 1. Furthermore, we confirm that Chrna2-expressing Martinotti cells selectively target L5 thick-tufted type A PCs but not thin-tufted type B PCs. Using optogenetic activation and inhibition, we demonstrate how Chrna2-Martinotti cells robustly reset and synchronize type A PCs via slow rhythmic burst activity and rebound excitation. Moreover, using optical feedback inhibition, in which PC spikes controlled the firing of surrounding Chrna2-Martinotti cells, we found that neighboring PC spike trains became synchronized by Martinotti cell inhibition. Together, our results show that L5 Martinotti cells participate in defined cortical circuits and can synchronize PCs in a frequency-dependent manner. These findings suggest that Martinotti cells are pivotal for coordinated PC activity, which is involved in cortical information processing and cognitive control.

## Author Summary

Cognitive functions and information processing are linked to the coordination of neuronal events and activities. This coordination is achieved through the synchronization of neuronal signals within subnetworks. Local networks contain different types of nerve cells, each of them playing distinct roles in the synchronization mechanism. To understand how synchronization is initiated and maintained, we have identified one of the key players using genetic strategies; we have identified a subtype of nicotine receptors uniquely expressed in cortical Martinotti cells. Because of their architecture and connection properties, Martinotti cells are able to synchronize ongoing activity of unconnected pyramidal cells (PCs). We show that this mechanism only applies to one subtype of PCs, thereby demonstrating that Martinotti cell inhibition is not spread randomly. By testing optimal firing patterns of Martinotti cells, we are able to coordinate the firing of this specific PC subtype over longer periods of time, showing how one unique interneuron is contributing to information processing.

## Introduction

Martinotti cells, ubiquitous to the cortex [1], are the most prominent cross-laminar interneuron subtype forming synapses in layer 1 onto the distal dendrites of cortical pyramidal cells (PCs) [1–3]. Despite this close structural relationship, the role of Martinotti cell inhibition is not clear. Studies identifying Martinotti cells by various markers have found different morphologies and microcircuit connectivity depending on the cortical layer in which their cell bodies reside [2]. In general, the division of neocortical interneurons into either parvalbumin-, somatostatin (SOM)-, or 5HT3aR-expressing cells [4–6] has been helpful for dissecting neural functionality; yet, these groups can be further subdivided and show partial overlap between interneuron markers. Martinotti cells are a subclass of SOM+ cells [7,2,4], and several combinations of transgenic lines have been created to try to genetically and morphologically isolate Martinotti cells [8–10]. For example, the SOM-cyclization recombinase (Cre) mouse line marks layer 1–projecting Martinotti cells with cell bodies in both layer 5 (L5) and layer 2/3 (infragranular and supragranular layers) but also labels non-Martinotti cells in layer 4 [10]. Although electrophysiologically, SOM+ Martinotti cells are often referred to as low-threshold spiking (LTS) neurons [3] or slow-inhibitory interneurons [11], early studies have shown up to four different firing patterns for Martinotti cells [2,9].

Functionally, cortical SOM+ interneurons have been suggested to provide a “blanket of inhibition” [12], a dense and nonspecific spread of inhibition on nearby PCs. Whether Martinotti cells are capable of generating such indiscriminate inhibition when firing simultaneously in large groups has not been tested. Martinotti cells that reside in the main cortical output L5 provide frequency-dependent disynaptic inhibition (FDDI) on neighboring PCs [13,14], an inhibitory mechanism that synchronizes two or more PCs by one or a few Martinotti cells [15]. Synchronized activities in the cortex have been reported in vivo [16] as well as in vitro, where slow oscillations appear to be initiated in L5 [17]. Moreover, computational studies suggest that Martinotti cell activity can synchronize L5 PC spiking through distal inhibition [18]; however, this has not been tested experimentally. It is intriguing that distal dendrite–targeting interneurons, generating attenuated inhibitory currents, can affect PC spike time output. Here, we genetically targeted the L5 Martinotti cell population using a nicotinic acetylcholine receptor α2 subunit (Chrna2)-Cre mouse line to investigate how Martinotti cell inhibition can synchronize L5 PC firing. Our results show that Chrna2-Cre–labeled L5 Martinotti cells were preferentially and reciprocally connected with thick-tufted PCs. Furthermore, we found that short burst firing of L5 Martinotti cells was able to reset L5 PC spiking and that controlling Martinotti cell activity to rapid bursts repeated in a slow rhythm was the most efficient inhibition to synchronize unconnected PCs. Finally, we show that L5 PC microcircuits could synchronize their own action potentials (APs) when coupled by L5 Martinotti cells and that inhibition was crucial for PC synchronization over prolonged periods.

## Results

### L5 Martinotti Cells Express the Nicotinic Acetylcholine Receptor Subunit Alpha 2

**Fig 1:**
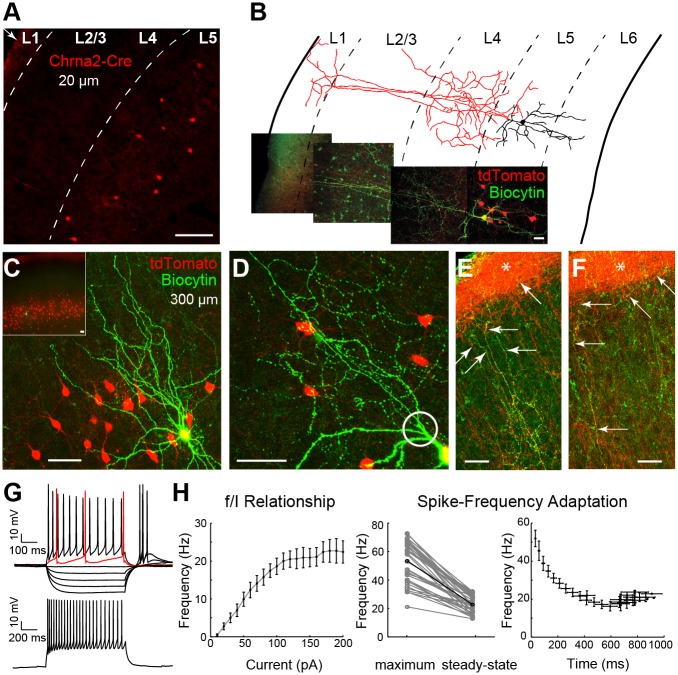
L5 Chrna2-Cre/*R26*^*tom*^ cells show Martinotti cell morphology and are low-threshold, slow accommodating firing. (A) Confocal image (20 μm, coronal slice) of primary auditory cortex of a *Chrna2-Cre/R26*^*tom*^ mouse showing tdTomato+ somas (red) in L5 with dense axonal arborizations in layer 1 (arrow in corner, scale bar = 100 μm). (B) Confocal image and tracing of a biocytin-filled tdTomato+ neuron (green). Reconstruction of soma and dendrites (black) and axon (red; scale bar = 20 μm) shows long axonal projections to layer 1. (C) Confocal images of a biocytin-filled (green) tdTomato+ neuron among several tdTomato+ neurons (red) show that cells have an ovoid cell body in L5, bipolar dendritic morphology, and proximal axonal arborizations. (D) Image illustrating how the *Chrna2-Cre/R26*^*tom*^ axons emerge from the main dendrite (circle). Scale bars = 50 μm. (E) Image showing the long axonal arborizations (arrows) from one biocytin-filled *Chrna2-Cre/R26*^*tom*^ cell (yellow) to layer 1 and the dense axonal ramifications (asterisk) in layer 1 from all *Chrna2-Cre/R26*^*tom*^ cells expressing tdTomato (red). Scale bars = 50 μm. (F) Example from another biocytin-filled *Chrna2-Cre/R26*^*tom*^ cell to emphasize axonal arborization extending laterally in layer 1, seen as a thin yellow axon at the border of the axonal plexus of *Chrna2-Cre/R26*^*tom*^ cell in layer 1. (G) *Top*: Example current clamp traces from a tdTomato+ cell showing low-threshold, accommodating firing (20 pA response in red, 100 pA in black, 500 ms) and rebound APs (−20 to −80 pA, 500 ms) typical for Martinotti cells. *Bottom*: Current clamp trace in response to a 200-pA, 1,000-ms-long stimulus used for analysis in (H). (H) *Left*: The frequency/current (f/I) curve of MCs^α2^ shows an average firing rate around 20 Hz (at 200 pA, 1,000 ms) indicating slow spiking properties. *Middle*: Difference in maximum frequency and steady-state frequency for each neuron to a 200 pA, 1,000-ms-long current step highlights an accommodating discharge. The black line depicts the mean adaptation. *Right*: Spike-frequency adaptation is shown as a function of time. Data (*n* = 36 cells) are presented as mean ± standard error of the mean (SEM) and shown in S1 Data.

To test whether Chrna2 can be used as a marker of Martinotti cells, we crossed Chrna2-Cre mice with a tdTomato reporter line (*R26*^tom^, Fig 1 and S1 Fig) [19,20]. L5 Chrna2-Cre/*R26*^*tom*^ cells were found in all cortices (S1A–S1C Fig, S1 Movie). Only very few Chrna2-Cre/*R26*^*tom*^ cells were detected in layer 2/3 (supragranular: 27 [2.4%] versus infragranular: 1102 [97.6%] cells in an 800-μm-thick section), suggesting that Chrna2-Cre/*R26*^*tom*^ specifically labels L5 neurons. Reconstructions of patched biocytin-filled Chrna2-Cre/*R26*^*tom*^ cells showed that 36 out of 37 (97.3%) cells met the criteria of deep layer Martinotti cells by having an ovoid cell body in L5, bipolar dendritic morphology, axons emerging from the main dendrite, proximal axonal arborizations, and long axonal projections to layer 1 with a dense arborization around PC distal dendrites (Fig 1B–1F and S2 Fig) [2,3,10,13]. The excluded cell had its cell body outside of L5. At higher magnifications, the axonal plexus of L1 is highlighted by red fluorescent signal (tdTomato) of Chrna2-Cre/*R26*^*tom*^ cell axonal ramifications (see star in Fig 1E, 1F and S1C, S1D and S2B Figs). Immunohistochemistry revealed that 30.3% of Chrna2-Cre/*R26*^*tom*^ cells were SOM+ (*n* = 3 mice, 2–3 mo old, 8 sections of 35-μm thickness, of a total of 792 cells (L1–L6); 297 cells were Chrna2+, 495 cells were SOM+, and 90 of these were double labelled for both genetically expressed tdTomato and SOM antibodies; S1D and S1E Fig). Counting cells only in L5, we found a total of 549 cells, of which 292 cells were Chrna2+, 257 were SOM+, and 85 of these were double labelled for both Chrna2 and SOM (29.1% of L5 Chrna2+ cells were SOM+; S1D and S1E Fig). Single-cell reverse transcription PCR (RT-PCR) of individually picked Chrna2-Cre/*R26*^*tom*^ cells (*n* = 7 cells, *n* = 2 animals) found 6/7 collected neurons to be positive for Glutamate decarboxylase 1 (GAD1; S1F Fig), while no cell was positive for the vesicular glutamate transporter subtype 1 or 2, indicating an inhibitory nature of Chrna2+ neurons. Membrane properties of Chrna2-Cre/*R26*^*tom*^ cells measured by whole-cell patch clamp revealed a mean input resistance of 337.28 ± 11.42 MΩ and a mean resting membrane potential of −63.69 ± 1.02 mV (*n* = 36 cells, S1 Data). The first AP generated upon 500 ms depolarizing current injections with 1-pA increments (on average, the first spike was reached in response to 18.11 ± 1.97 pA) had a mean AP amplitude of 72.61 ± 2.20 mV, an AP threshold of −43.28 ± 0.53 mV, an AP half-width of 1.92 ± 0.08 ms, and a first spike latency of 254.75 ± 24.24 ms (*n* = 36 cells, S1 Data). Afterhyperpolarizations (AHPs) measured after the first AP had a mean magnitude of −8.28 ± 0.72 mV with a gradual depolarization over repeated spikes, and each AHP displayed both a fast and a slow component (Fig 1G, S1 Data) [2]. Hyperpolarizing currents generated rebound afterdepolarizations (ADPs) and, on average, 2.50 ± 0.25 rebound APs (at −80 pA, 500 ms) upon termination of current steps but did not produce a sizable membrane “sag,” suggesting that these cells have a minimal hyperpolarization-activated current (I_h_, Fig 1G, top, S1 Data). The Chrna2-Cre/*R26*^*tom*^ cell firing frequency versus current relationship showed a linear increase of average firing rate with increasing current towards a frequency of 22.4 ± 2.49 Hz (at 200 pA, *n* = 36 cells; Fig 1H, left, S1 Data), indicating that Chrna2-Cre/*R26*^*tom*^ cells are slow spiking interneurons. The relationship between maximum frequency (52.87 ± 2.44 Hz, *n* = 36 cells, S1 Data) and steady-state frequency (21.87 ± 1.02 Hz, *n* = 36 cells, S1 Data) revealed a spike-frequency adaptation ratio (see Materials and Methods) of ~59% in response to a 200-pA step (Fig 1G, bottom and Fig 1H, middle). The spike-frequency adaption shows how firing frequency of Chrna2-Cre/*R26*^*tom*^ cells decreases as a function of time (20.16 ± 2.44 Hz at 416 ms, *n* = 36 cells; Fig 1H, right). In summary, both morphological and electrophysiological characteristics of infragranular Chrna2-Cre/*R26*^*tom*^ cells (~97%) are similar to those reported in previous studies of Martinotti cells [2,13,21], thus we hereafter refer to these L5-specific Chrna2-Martinotti cells as MCs^α2^.

### MCs^α2^ Are Reciprocally Connected with Thick-Tufted Type A PCs

**Fig 2:**
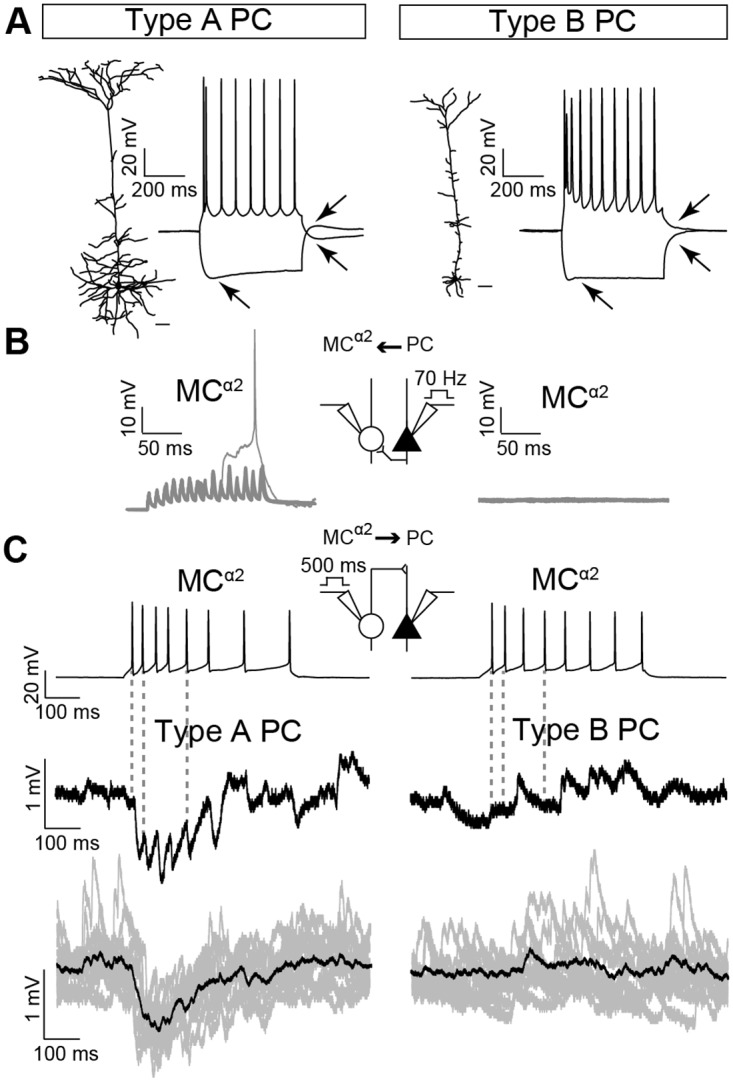
MCs^α2^ connect to local type A PCs but not type B PCs. (A) *Left*: The reconstruction of a typical type A PC showing a thick-tufted dendrite (scale bar = 40 μm) and its response to a 500-ms-long depolarizing (100 pA) and hyperpolarizing (−60 pA) stimulus. *Right*: A representative type B PC with a thin-tufted apical dendrite (scale bar = 40 μm) and its current clamp response (as for *left*). Note the deeper AHP (following a depolarizing current pulse), the more prominent sag (during a hyperpolarizing current pulse), as well as the pronounced rebound ADP (following a hyperpolarizing current pulse) in the type A PC compared to type B PC (see arrows). (B) Type A PCs can excite postsynaptic MCs^α2^ (*inset*) and generate facilitating EPSPs (*left*, *n* = 7/9 pairs, 12 repetitions from one example pair are shown) when stimulated with high frequency (70 Hz), whereas type B PCs do not trigger EPSPs in MCs^α2^ (*right*, *n* = 0/9 pairs, 12 repetitions). *Inset* shows experimental setup. (C) Typical MC^α2^ discharges (*top*) to a 500-ms-long (25 pA) stimulus are shown. *Inset* shows experimental setup. MC^α2^ spikes cause inhibition in postsynaptic type A PCs (*inset*) displaying synaptic depression (*middle left*, *n* = 7/9 pairs), whereas type B PCs do not receive MC^α2^ inhibition (*middle right*, *n* = 0/9 pairs). Grey dashed lines highlight timing of presumably individually generated IPSPs for type A PCs, whereas for type B PCs, dashed line shows the lack of response. Example IPSP responses of 12 repetitions are shown in grey, mean response in black (*bottom*).

Previous studies have speculated that SOM+ cells (including Martinotti cells) predominantly contact specific subpopulations of PCs [21,22]. Thus, we patched pairs of a MC^α2^ and its neighboring PC in L5 (≤60 μm). Next, we categorized PCs into type A and type B cells based on morphological and electrophysiological criteria [23]. Cells with a large cell body, thick-tufted basal dendrites with apical dendrites extensively branching in layer 1, burst-regular spiking, responding with large AHPs, prominent hyperpolarization sags, and pronounced rebound ADP were classified as type A PCs (Fig 2A, left). Cells with small soma, thin-tufted basal dendrites with limited spreading apical dendrites, absence of AHP or ADP, and small hyperpolarization sags were classified as type B PCs (Fig 2A, right). We expected L5-specific MCs^α2^ to be locally connected to PCs [2,13] and found, amongst morphologically reconstructed pairs of patched cells, that 77% of type A PCs–MCs^α2^ were connected (*n* = 7/9), while none of the patched type B PCs–MCs^α2^ were connected (*n* = 0/9). Out of the paired MC^α2^–type A PC recordings, 55% (*n* = 5/9) of pairs were reciprocally connected. Paired recordings of PCs and MCs^α2^ revealed that high-frequency stimulation (70 Hz) of type A PCs generated excitatory postsynaptic potentials (EPSPs), or an occasional spike, in MCs^α2^ (example shows 12 repetitions from one patched type A PC–MC^α2^ pair; Fig 2B, left), whereas type B PC stimulation did not result in EPSPs in local MCs^α2^ (12 repetitions; Fig 2B, right). Additionally, inhibitory postsynaptic potentials (IPSPs) were generated in type A PCs (IPSP amplitude: −1.08 ± 0.12 mV; example shows 12 repetitions from one patched MC^α2^–type A PC pair; Fig 2C, left) after MC^α2^ stimulation, whereas no inhibitory responses were observed in type B PCs (12 repetitions; Fig 2C, right). Thus, our data suggest that MCs^α2^ connect with type A PCs and not type B PCs.

### Optogenetic Activation of MCs^α2^ Shows Frequency-Dependency of MC–PC Inhibition

**Fig 3:**
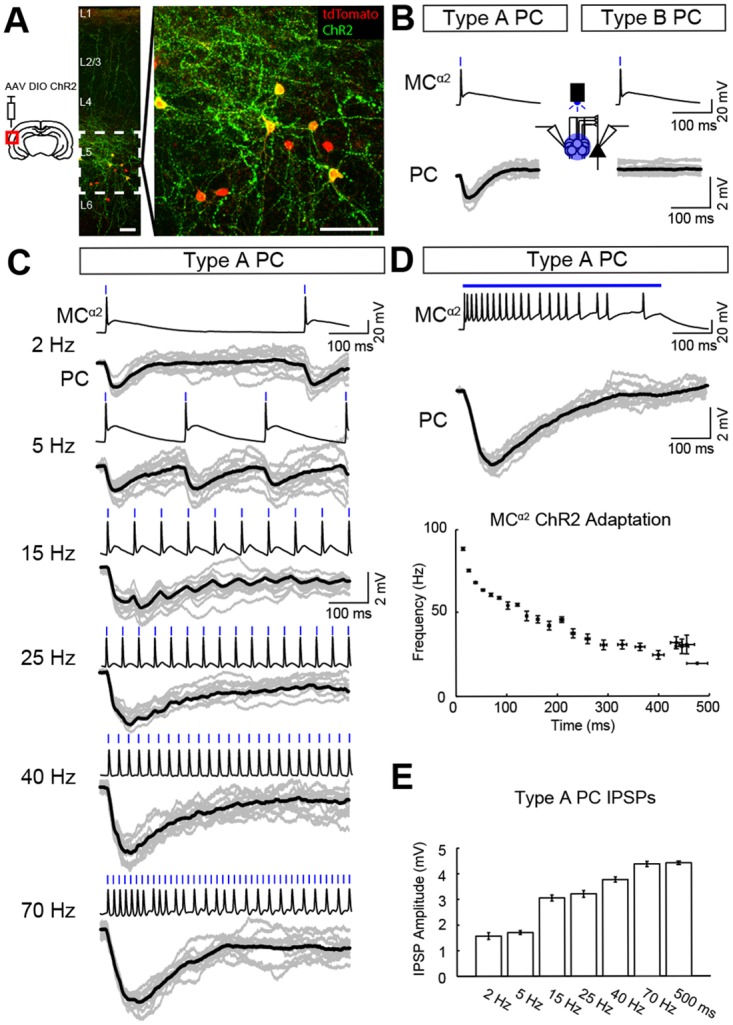
MC–PC inhibition is frequency dependent. (A) Expression of AAV-DIO-ChR2-EYFP (green) in MCs^α2^ (red) in a primary auditory cortical slice used for optogenetic stimulation, with inset on L5 showing overlap of membrane expression in yellow (scale bars 50 μm). (B) Optogenetic activation (3-ms light pulses, 488 nm) of a group of MCs^α2^ induced IPSPs in type A PCs (*left*) but not type B PCs (*right*) (*n* = 12 cells, single examples in grey, mean in black). (C) Example traces show MCs^α2^ responses to blue light stimulation at various frequencies (3-ms blue light pulses at 2, 5, 15, 25, 40, and 70 Hz) and the corresponding IPSPs in a nearby type A PC (*n* = 12 cells, single examples in grey, mean in black). At higher frequencies (≥15Hz), the MC^α2^–PC synapse showed depression. Note that MCs^α2^ could not follow 70-Hz light stimulation for prolonged time. (D) *Top*: Continuous light stimulation of MCs^α2^ (500 ms) generated large type A IPSP amplitudes (*middle*; *n* = 12 cells, single examples in grey, mean in black) similar in magnitude to IPSPs generated by high-frequency stimulation at 70 Hz. *Bottom*: Spike-frequency adaptation of MCs^α2^ is shown as a function of time (see also S3B–S3D Fig). (E) Mean IPSP amplitudes in type A PCs following stimulation of MCs^α2^ at different frequencies (from (C) and (D); 2 Hz: −1.57 ± 0.13 mV, 5 Hz: −1.70 ± 0.08 mV, 15 Hz: −3.05 ± 0.12 mV, 25 Hz: −3.20 ± 0.13 mV, 40 Hz: −3.76 ± 0.10 mV, 70 Hz: −4.37 ± 0.10 mV, 500 ms: −4.41 ± 0.07 mV; 2, 5 Hz versus 15, 25 Hz ≙ *p* < 0.0001; 15, 25 Hz versus 40 Hz ≙ *p* < 0.0001; 40 Hz versus 70 Hz, 500 ms ≙ *p* < 0.001; mean ± SEM, ANOVA, *n* = 12 cells, S3 Data).

We next investigated the influence of MC inhibition on PCs when simultaneously activating a large group of MCs^α2^ in Chrna2-Cre mice (1–2 mo old) previously injected with floxed Channelrhodopsin-2 (ChR2; Fig 3A). Compared to electrical stimulation of single MCs^α2^, light activation of MC^α2^ groups produced IPSPs in type A PCs with a higher mean amplitude (from −0.96 ± 0.05 mV to −1.41 ± 0.04 mV), a smaller mean time to peak (from 29.53 ± 1.24 ms to 20.54 ± 0.97 ms), and a decreased mean half decay time (from 63.46 ± 2.04 ms 51.20 ± 2.08 ms [all comparisons: *n* = 12 cells; a total of 54 IPSPs, *p* < 0.001, Fig 3B, left and S3A Fig, S2 Data]). We also recorded from type B PCs (*n* = 12 cells), but no IPSPs were observed in type B PCs in response to blue light stimulation of ChR2+ MCs^α2^ (Fig 3B, right). We next tested different stimulation frequencies (2, 5, 15, 25, 40, and 70 Hz) [13,18] for ChR2+ MCs^α2^ to investigate the role of MC^α2^ firing frequency on IPSP amplitude in type A PCs (*n* = 12 cells; Fig 3C). We found a nonlinear relationship between IPSP amplitude and MC^α2^ stimulation frequency in which, at higher frequencies (>15 Hz), IPSPs summed into smooth compound IPSPs, probably due to the depressing synaptic properties of the MC^α2^-to-PC connection [13]. To further characterize the frequency-dependency of the MC^α2^–PC IPSPs, we stimulated MCs^α2^ with continuous light, which generated accommodating firing in MCs^α2^ (Fig 3D, bottom and S3B–S3D Fig) but still large IPSPs in PCs (*n* = 12 cells, Fig 3D and 3E, S3 Data). This suggests that large compound IPSPs can be generated in type A PCs when MCs^α2^ fire at high frequencies and that the compound IPSP amplitude mainly depends on the firing frequency during the first 300 ms (Fig 3D and 3E).

### Bursts of MCs^α2^ Can Reset Type A PC Firing

**Fig 4:**
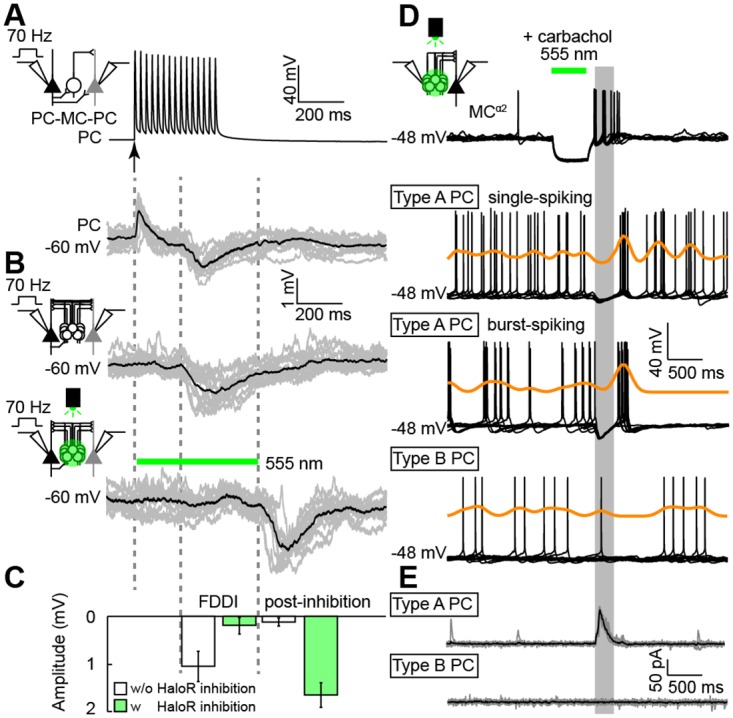
MCs^α2^ contribute to FDDI, and MC^α2^ burst firing can reset type A PC spikes. (A) High-frequency stimulation (70 Hz, see arrow) of a presynaptic PC (▲) generates delayed IPSPs on a neighboring PC (▲) via intermediate MCs^α2^ (О). A mixed excitation (due to a monosynaptic PC–PC connection) followed by a disynaptic inhibition is shown (*n* = 12 cells, single examples in grey, mean in black). (B) An example of disynaptic inhibition alone (*top*) is shown (*n* = 12 cells, single examples in grey, mean in black). Silencing of HaloR-expressing MCs^α2^ via green light (555 nm) prevents FDDI, although IPSPs are generated following termination of green light stimulation (*bottom*, *n* = 12 cells, single examples in grey, mean in black). (C) Mean IPSP amplitudes with (white) and without (green) FDDI at two different time points. (D) Responses from HaloR-expressing MCs^α2^ (*top*) and local type A PCs (single-spiking and burst-spiking; *middle*) and type B PCs (*bottom*) are shown in presence of carbachol (10 μM). Green light stimulation (500 ms) hyperpolarizes HaloR-expressing MCs^α2^ and upon termination MC^α2^ rebound APs are triggered. This burst of APs generates robust inhibition in local postsynaptic type A PCs that synchronizes the timing of PC (rebound) APs. Kernel density estimates (orange) highlight increased (peaks) and decreased (valleys) co-occurance of APs. (E) Example of voltage clamp responses for type A (*top*) and type B (*bottom*) PCs in response to MCs^α2^ burst firing (single examples in grey, mean in black). Values are shown in S4 Data.

Martinotti cells have been shown to provide FDDI [13,24,15] onto PCs. To examine whether L5-specific MCs^α2^ generate FDDI, we patched pairs of type A PCs with cell bodies next to MCs^α2^ and provided high-frequency (70 Hz) current injection to one PC (Fig 4A, top). This led to an early EPSP in the other PC, presumably due to monosynaptic PC–PC connections, followed by a delayed inhibition (FDDI, amplitude: −1.02 ± 0.23 mV, time to peak: 57.16 ± 2.43 ms, half decay time: 95.34 ± 5.64 ms, *n* = 12 cells; Fig 4A, bottom, S4 Data). In some cases, patched type A PC pairs were not monosynaptically connected and only the FDDI was observed (amplitude: −1.05 ± 0.21 mV, time to peak: 91.33 ± 2.83 ms, half decay time: 123.34 ± 5.87 ms, *n* = 12 cells; Fig 4B, top and Fig 4C, S4 Data). To confirm that the FDDI response was mediated by MCs^α2^, we silenced MCs^α2^ in slices from Chrna2-Cre/Halorhodopsin (HaloR)-floxed mice (1–2 mo old) with green light. Indeed, green light abolished the delayed inhibition (*n* = 12 cells; Fig 4B, bottom and Fig 4C, S4 Data). However, we also noted that large IPSPs occurred in both patched type A PCs upon termination of the light pulse (amplitude: −1.66 ± 0.16 mV, time to peak: 53.45 ± 2.23 ms, half decay time: 88.45 ± 4.53 ms, *n* = 24 IPSPs, all comparisons: *p* < 0.001; Fig 4B, bottom and Fig 4C, S4 Data). Current clamp recordings from MCs^α2^ showed that the green light generated strong hyperpolarization of MCs^α2^ and that, subsequently, bursts of rebound spikes were generated in HaloR-expressing MCs^α2^ upon light termination (Fig 4D, top and S4A and S4B Fig, S5 Data). HaloR-activation for 500 ms consistently evoked one or more rebound APs with varying frequency in MCs^α2^ that could not be blocked by the I_h_ blocker ZD7288 (20 μM, S4C Fig), similar to I_h_-independent rebound APs in distal dendrite–targeting X98 cells [9]. Additionally, MCs^α2^ and type A PCs were patched in the presence of carbachol (10 μM) to further examine how MCs^α2^ could modulate L5 PC spontaneous firing. Carbachol depolarized PCs and MCs^α2^ by 10 to 15 mV and did not result in a specific oscillatory frequency as seen with high concentrations of carbachol; instead, it showed broad peaks in the power spectral density plots (S5 Fig). Two firing patterns could be distinguished for type A PCs [25–28]: single-spiking (*n* = 42 cells; Fig 4D, middle) and burst-spiking (*n* = 18 cells; Fig 4D, middle). Independent of the type A PC firing type, bursts of HaloR-induced rebound spikes from MCs^α2^ caused large, compound IPSPs (S6 Fig, S6 Data) that resulted in a resetting of type A PC firing (Fig 4D, middle). We define resetting as temporally aligning spiking after a period of inhibition. APs from type A PCs aligned around 500 ms (single-spiking: 556.19 ± 9.42 ms, *n* = 42 cells; burst-spiking: 524.72 ± 10.17 ms, *n* = 18 cells, S4 Data) after light-off for HaloR-inhibition of MCs^α2^. The shape of the summed type A PC IPSP trace corresponded well to the first 3–4 rebound APs of MCs^α2^ (S6A Fig) and steadily evoked subsequent post-inhibitory rebound APs in the type A PCs (*n* = 60 cells; Fig 4D, middle) but not type B PCs (*n* = 12 cells; Fig 4D, bottom). Note that the IPSP amplitude more than doubled with carbachol present (V_m_ = −60 mV; IPSP amplitude = −1.66 ± 0.26 mV, Fig 4B, bottom; V_m_ = −48 mV [single-spiking PC]; IPSP amplitude = −3.51 ± 0.48; V_m_ = −48 mV [burst-spiking PC]; IPSP amplitude = −6.30 ± 0.91 mV, all comparisons: *p* < 0.0001; S6B and S6C Fig, S6 Data). Voltage clamp experiments (holding at −60 mV) further highlighted the presence of large IPSCs in type A PCs (107.40 ± 3.54 pA, *n* = 3 cells; Fig 4E, top, S4 Data) and the absence of IPSCs in type B PCs (*n* = 3 cells; Fig 4E, bottom). Together, these results show that a rapid burst of MC^α2^ APs can abruptly halt the firing of type A PCs and also reset PC firing by temporal coupling rebound APs of PCs while leaving type B PC firing unaffected. Therefore, we only aimed for type A PCs for the remainder of the study.

### Bursts of MC^α2^ APs Synchronize Firing of Type A PCs at Slow Frequencies

**Fig 5:**
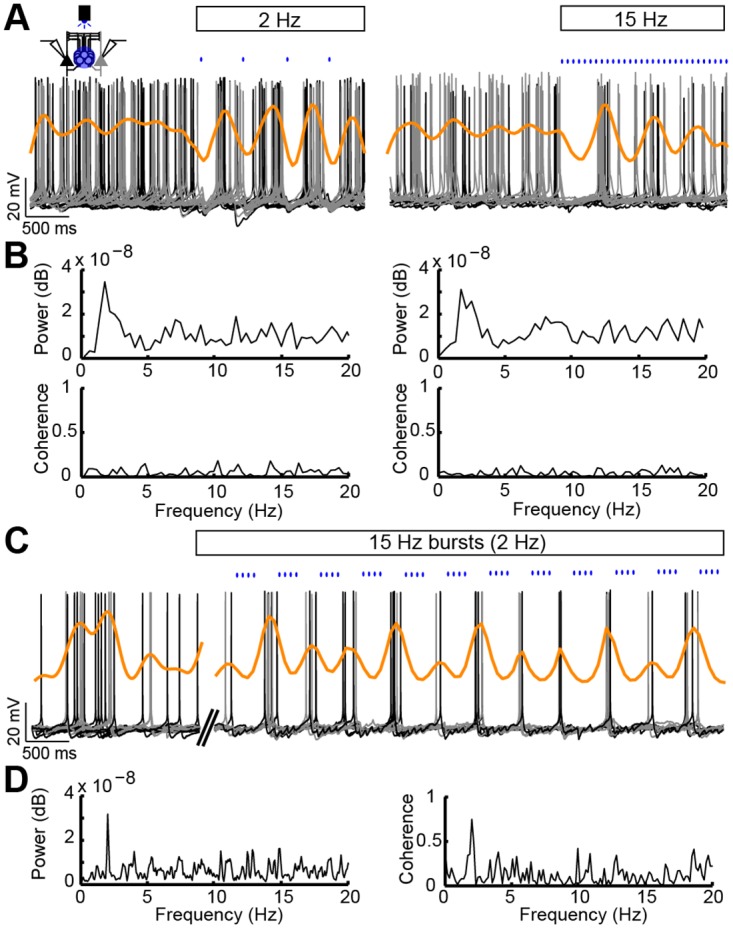
MC^α2^ bursts synchronize type A PC firing in slow frequencies. (A) Population response of 12 dual recordings of unconnected type A PCs (*n* = 24 cells; 12 black and 12 grey PC spike trains) before and during pulsed light stimulation of ChR2-expressing MCs^α2^ (2 Hz [*left*] and 15 Hz [*right*]). Kernel density estimates (orange) show increased (peaks) and decreased (valleys) co-occurrences of APs. (B) Mean power spectral density plots for both cases revealed peaks around 2 Hz (*top*) but flat mean coherence plots (*bottom*), suggesting no synchronization (correlation) between firing of type A PCs within a frequency range of 0–20 Hz (*n* = 24 cells). (C) Pairwise overlayed type A PC voltage traces are shown in response to combined light stimulation (i.e., 15-Hz bursts in 2 Hz). Kernel density estimates (orange) highlight co-occurring PC APs. (D) Mean power spectral density plot (*left*) and mean coherence plot (*right*) (from [C], *n* = 24 cells), show a peak at 2 Hz corresponding to the rhythmic activation of MCs^α2^. Peak values are shown in S7 Data.

It is unknown how PCs synchronize their firing, although computational studies have suggested a role for distal dendrite–targeting interneurons in synchronizations [11,18,29]. Thus, we only patched unconnected type A PCs and recorded spontaneous firing, in the presence of carbachol, from two PCs simultaneously (*n* = 24 cells) while optogenetically stimulating the MC^α2^ population at various frequencies (2, 5, 15, 25, 40, and 70 Hz). To identify the frequency of MC^α2^ activity that best temporally aligns unconnected, randomly firing, type A PCs, we recorded repeats of 4-s sweeps (2 s light-off, 2 s light-on). When pairwise superimposing simultaneous recordings from two type A PCs, we observed that light stimulation of 2 Hz or 15 Hz (*n* = 24 cells; 12 black and 12 grey PC spike trains; Fig 5A) created a rhythmical firing pattern of type A PCs highlighted by kernel density estimates, which show the distribution of APs over time (orange traces). Although mean power spectral density plots from both 2 Hz and 15 Hz MC^α2^ stimulation revealed peaks around 2 Hz (1.99 ± 0.09 versus 1.87 ± 0.14 Hz, *n* = 24 cells), other frequencies tested (5, 25, 40, and 70 Hz) did not result in any clear peaks (Fig 5B, top and S7 Fig, S7 Data). This indicates that MCs^α2^ preferentially give rise to slow frequencies in a group of type A PCs. However, flat mean coherence plots of pairwise analyzed PCs did not show any correlation between simultaneously recorded type A PCs in specific frequency bands plotted up to 20 Hz. This suggests that type A PCs as a population can produce an oscillatory firing rhythm, but individual cells are mostly out of phase and not synchronized with each other (*n* = 24 cells; Fig 5B, bottom). To generate complete synchrony between type A PCs, we hypothesized that a rapid burst of MC^α2^ activity could reset/align type A PC spiking (3–4 APs as seen in Fig 4D and S6A Fig) and a slow rhythm could maintain in-phase synchronous firing. To test this, we patched two unconnected type A PCs and stimulated MCs^α2^ with 15-Hz bursts every 500 ms (2 Hz). This stimulation protocol resulted in high AP synchronization (Fig 5C) directly after MCs^α2^ were paused. Mean power spectral density (peak at 2.02 ± 0.04 Hz, *n* = 24 cells) and mean coherence examination showed that type A PCs followed MC^α2^ stimulation frequency and were pairwise aligned in that frequency (Fig 5D, S7 Data), suggesting synchronized firing of type A PCs at slow frequencies. This shows that MCs^α2^ have means to both initiate and maintain prolonged type A PC synchronous firing.

### Slow Frequency Burst Stimulation of MCs^α2^ Is Synchronizing Type A PC Spike Timing via Minimally Depressing IPSPs

**Fig 6:**
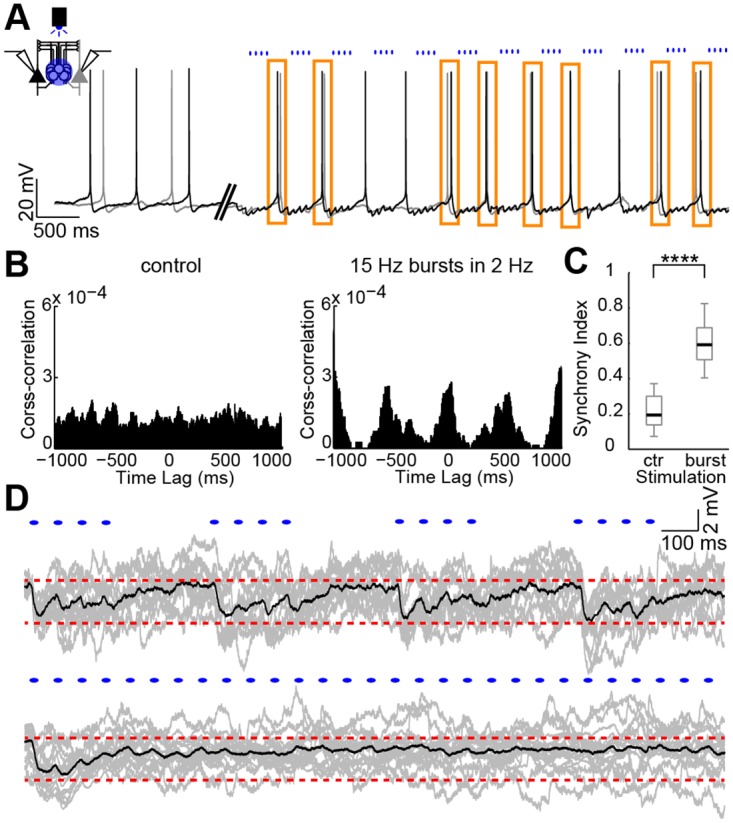
Repeated bursts of MC^α2^ inhibition synchronize type A PC spike trains via minimally depressing IPSPs. (A) Voltage traces from an unconnected pair of type A PCs (black and grey) with MCs^α2^ stimulated in 15-Hz bursts at 2 Hz (blue dots). Orange rectangles highlight synchronous APs. (B) Mean cross-correlograms (*n* = 24 cells) show little synchrony before light stimulation and increased synchrony during light stimulation (15-Hz bursts in 2 Hz) of MCs^α2^ as shown by a prominent peak around zero (and recurring peaks at every 500 ms). (C) Box plots of the synchrony indices for control and 15-Hz bursts show the significant increase of synchrony (0 ≙ no synchronization, 1 ≙ full synchronization) when MCs^α2^ are stimulated by blue light in brief bursts (*n* = 12 dual recordings, *p* < 0.0001, two-tailed Student’s paired *t* test). Values are shown in S8 Data. (D) IPSPs in type A PCs (*n* = 24 cells, single examples in grey, mean in black) following burst protocol of 15-Hz stimulation in 2 Hz (top) and constant 15-Hz light stimulation (bottom). Note minimal-depressing inhibition in the top and the depression of IPSPs leading to a rapid diminution of inhibition in the bottom (red dashed lines for improved visualization).

Next, we sought to quantify the synchrony (provide a synchrony index [30]) between type A PC firing when MCs^α2^ were activated in bursts of 15 Hz. A representative recording of two simultaneously captured type A PCs is shown in Fig 6A, where initially unsynchronized type A PCs aligned during MC^α2^ stimulation (orange rectangles highlight synchronized APs). In the absence of MC^α2^ stimulation, the mean cross-correlograms of pairwise analyzed recordings showed only low magnitude peaks, while light stimulation organized firing of both type A PCs in cohorts every 500 ms (*n* = 24 cells; Fig 6B). A 3-fold increase in the synchrony index could be extracted from the cross-correlograms when MCs^α2^ were light stimulated (control: 0.21 ± 0.03, burst stimulation: 0.61 ± 0.04, *n* = 12 dual recordings, *n* = 24 cells, *p* < 0.0001; Fig 6C, S8 Data). Thus, we conclude that delivery of MC^α2^ inhibition in bursts of 15 Hz indeed synchronized type A PC firing. This is likely due to burst firing repeated in slow frequency, causing inhibition in type A PCs with little depression compared to continuous 15 Hz stimulation of MCs^α2^ showing apparent synaptic depression (examples in grey, mean in black, red dashed lines for visual guidance, Fig 6D). Interestingly, the 15-Hz continuous light stimulation revealed that bursting PCs can switch firing patterns from burst-spiking into single-spiking [25,18] during continuous low-magnitude inhibition (S8A Fig). This change in firing was observed only at near-threshold potentials; the physiological role remains to be studied (S8B Fig).

### Type A PCs Can Auto-Synchronize Their APs When Intercoupled by MCs^α2^

**Fig 7:**
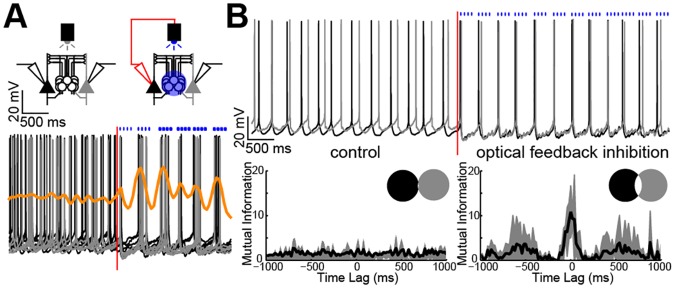
Type A PCs auto-synchronize via MC^α2^ inhibition. (A) Coupling one PC (black) out of two unconnected type A PCs to the light source/optical feedback inhibition system shows unsynchronized activity before and synchronized APs during optical feedback inhibition. A total of 24 PC discharges pairwise aligned to the first PC AP with optical feedback inhibition are shown (*n* = 24 cells; 12 black and 12 grey spike trains). Kernel density estimates (orange) highlight increased (peaks) and decreased (valleys) co-occurrence of APs. Note that the time points of the blue light depend on the PC APs during the optical feedback inhibition and therefore vary between PC pairs. (B) *Top*: One pair of simultaneously recorded unconnected type A PCs (black and grey) showing discharges before and during the optical feedback inhibition. *Bottom*: Pairwise mutual information index versus time lag from recordings in (A) showing low mutual information for unconnected PCs and high mutual information around 0-ms lag for PCs coupled by optical feedback inhibition (*n* = 12 dual recordings, 24 cells). *Inset*: Amount of overlap in Venn diagrams (black and grey circles) shows low mutual information for unconnected (*left*) and significantly higher mutual information for coupled (*right*) PCs (*p* < 0.05, two-tailed Student’s paired *t* test). Values are shown in S9 Data.

To test if type A PC circuits can self-synchronize their firing through Martinotti cell activation, we designed a closed-loop system (optical feedback inhibition [31]) for paired recordings that delivered four blue light pulses (15 Hz) to the MCs^α2^ when one PC fired in the presence of carbachol (*n* = 24 cells; 12 black and 12 grey PC spike trains; Fig 7A, inset) in tissue from Chrna2-Cre mice previously injected with floxed ChR2. Kernel density estimates showed increased co-occurrences of type A PC APs during optical feedback inhibition (Fig 7A). Moreover, because these experiments involved a leading and a following type A PC, it was possible to calculate the statistical dependency between the spike trains of two PCs and to express this with a mutual information index (see Materials and Methods). This index gives an estimate of how well one signal can predict the other and is helpful to interpret to what extent one PC can drive another, e.g., via recurrent or feedforward inhibition. In controls, the mutual information index was low (2 ± 1, *n* = 12 dual recordings), whereas turning on the optical feedback inhibition resulted in immediate auto-alignment of type A PC APs with an increased index (11 ± 5, *n* = 12 dual recordings, *p* < 0.05; Fig 7B, S9 Data). Venn diagrams show the mutual information as the degree of overlap between two circles, representing each PC train as entropy (Fig 7B, inset). The overlap demonstrates the predictive value (mutual dependency) between a known PC train and a following PC train. When shifting one spike train relative to the other, the incremental mutual information index plot (mutual information index as a function of time lag, Fig 7B) showed that the mutual dependency was largest around 0-ms lag, suggesting high synchronization of the two PCs directly when coupled by optical feedback inhibition. Peaks around ± 400–600 ms indicate that this activity-dependent inhibition causes repeated synchronization every 400–600 ms.

## Discussion

We found that MCs^α2^ were exclusively synaptically connected to large, thick-tufted PCs, often referred to as L5B PCs [32] or type A PCs [23]. Different PC morphologies seem to be associated with different connectivity patterns in the brain, e.g., large, thick-tufted PCs are usually synonymously named subcerebral projection neurons or pyramidal tract neurons, whereas thin-tufted PCs, or type B PCs [23] are callosal projection neurons or intratelencephalic neurons [26–28]. Our in vitro preparation could not define PCs according to connectivity patterns; however, based on the extensive branching of the distal dendrites and the large triangular-shaped cell bodies, we find it likely that the type A PCs correspond to the thick-tufted PCs [22] and are probably subcortically projecting [26–28]. Thick-tufted type A PCs can further be described by firing properties as single-spiking or burst-spiking [25–28]. Typically, at near-threshold potentials, bursting cells respond with two or more bursts, of two or more APs, generated in quick succession with short interspike intervals [25]. Burst properties of PCs disappear with increasing current injections [25] and may be dependent on the size of the dendritic tree [33]. In addition to morphological variances, such as a smaller soma compared to type A PCs, type B PCs had characteristic electrophysiological differences. Our pair-recordings between type A PCs and MCs^α2^ confirmed that type A PCs provided facilitating synaptic responses in MCs^α2^. We also found depressing synaptic connections from MCs^α2^ to type A PCs [13], while no type B PC connectivity with MCs^α2^ was observed. Lack of IPSPs in type B PC was not likely due to shunting of inhibition, as voltage clamp recordings also failed to find synaptic connectivity between MCs^α2^ and type B PCs. However, due to difference in thin- versus thick-tufted morphology, it is possible that the internal solution creates less dialysis of the chloride ion Cl^-^ concentration in type B PCs compared to type A PCs. Therefore, perforated patch recordings would be needed to firmly rule out the possibility of shunting of inhibition. Still, we found that FDDI, a Martinotti cell–dependent feature [13,24,15] was relayed by MCs^α2^ and consequently was specific for type A PCs. This is in agreement with a previous study showing FDDI between thick-tufted PCs but not between corticocallosally projecting cells [22].

Distal inhibition by individual MCs is important for shaping local dendritic voltage–activated responses. FDDI combined with dendritic depolarization has shown that MCs can attenuate back-propagating AP-activated Ca^2+^ spike firing and thereby reduce burst firing of PCs [34]. On the network level, collective and precisely timed Martinotti cell activity can further be potent enough to affect somatic spike generation. Here, we first used HaloR to examine if blocking MC^α2^ activity could eliminate the appearance of FDDI in thick-tufted PCs [13,24,15]. This led to the observation that on the termination of green light MCs^α2^ fired bursts of HaloR-induced rebound spikes, inhibiting PCs and subsequently causing hyperpolarization-induced rebound APs that could reset PC firing. The HaloR-induced rebound in MCs^α2^ is a methodological artifact and has little physiological relevance; however, it is interesting to speculate whether Martinotti cells receive inhibition that could generate rebound spikes. Recently, the vasoactive intestinal peptide (VIP) interneuron has been shown to densely inhibit Martinotti cells [35]. The high connection probabilities between VIP cells and Martinotti cells [36] suggest that VIP cells could provide strong hyperpolarization in Martinotti cells for the possible generation of rebound excitation. Rebound spikes have been previously demonstrated to occur in entorhinal cortex neurons in vivo and are attributed to play a role in generating grid cell fields that usually arises when grid cells fire synchronized [37,38]. A similar role could be applicable to rebound spikes in the neocortex, where a “blanket of inhibition” [12] evolves through the synchronized spread of inhibition, serving to coordinate PC firing. Thereby, VIP cells appear to make "holes in the blanket of inhibition" [35] by inhibiting Martinotti cells [35,36]. In other words, VIP cell activity might regionally disrupt coordinated PC firing while local Martinotti cell activity could reset and rescue PC synchronous firing.

Second, focusing on the combined activity of ChR2-expressing MCs^α2^, our data show that bursts of MCs^α2^ were able to reset type A PC firing and, if repeated, could synchronize PC activity. In a computer model, oscillatory inhibition of the distal PC dendrite at 10–20 Hz, presumably by LTS SOM+ Martinotti cells, was shown to control L5 PC firing [18]. Our findings support that 15 Hz firing of MCs^α2^ can align type A PC firing but also show that 15 Hz firing in short bursts more reliably synchronizes PCs compared to continuous 15 Hz firing. In other computational models, the importance of a beta rhythm in regulating gamma oscillations and intercortical signaling has been demonstrated and, furthermore, that the beta frequency is regulated by cholinergic modulators [11,29,39]. In this respect, the exclusive expression of the alpha 2 cholinergic receptors in MCs^α2^ is noteworthy and may suggest a specific role for MCs^α2^ in transmitting the modulatory action of cholinergic signaling. Cholinergic modulation of LTS cells has been suggested to generate beta oscillatory activity (beta2) in L5 of the primary auditory cortex [40]. These oscillations were insensitive to the muscarinic antagonist atropine but sensitive to the nicotinic receptor antagonist d-Tubocurarine [40]. Thus, computational and experimental studies indicate that the beta rhythm is important for network properties [40,41]; however, beta activity in bursts repeated in slow frequency has not been reported previously. At this slow frequency MC^α2^–PC inhibition shows minimal depression, similar to the minimal depression of slow firing SOM+ interneurons defined by their green fluorescent protein expression in a transgenic mouse (GIN-cells) [42], and therefore, a combination of rapid bursting and slow rhythmical inhibition seems most effective to synchronize PCs.

Genetic targeting and optical feedback inhibition are a potent technique to study how PCs can drive a population of interneurons by their innate rhythm. A previous study used a closed-loop system to optogenetically produce feedback inhibition onto PCs from parvalbumin+ interneurons [31]. Sohal et al. used synthetic excitatory post synaptic currents (EPSCs, dynamic clamp) in a single PC triggering parvalbumin+ interneuron excitation with light [31]. Differently, in our study, we depolarized optically stimulated MCs^α2^ in the presence of carbachol and measured synchronization of simultaneously recorded PCs using mutual information. Analogously to our optogenetic stimulation, gap junctions could provide a physiological mechanism for the synchronization of interneuron populations [42–44]. Berger et al. have shown the existence of electrical coupling between L5 MCs [15]. It will be interesting in the future to explore the existence of gap junctions between MCs^α2^.

The Chrna2-Cre/*R26*^*tom*^ mouse line simplifies identification and characterization of L5 Martinotti cells. MCs^α2^ are morphologically and electrophysiologically homogenous, further evincing the specificity of our marker. The dense axonal plexus observed in L1 and the near absence of Cre+ cell bodies in L2 (2.4%) in Chrna2-Cre/*R26*^*tom*^ mice also indicate that Cre+ cells are, in fact, L5 MCs. Still, we found a high proportion of Cre+ cells in Chrna2-Cre/*R26*^*tom*^ mice that were not labelled with the antibody against SOM, and this could be due to extra-somatic location of the peptide. So far, SOM-Cre is the most widely used transgenic mouse line for targeting MCs together with the GIN mouse [8,45], but still SOM-Cre has been shown to label all cell layers [46]. Here, we provide a layer-specific, single genetic marker for MCs across the cortex and confirmed their inhibitory nature using single cell RT-PCR. Although we did not explicitly block optogenetically evoked, inhibitory postsynaptic currents of MCs^α2^ (e.g., with Gabazine), Martinotti cell dendritic inhibition in vivo has been shown to be GABA_A_-mediated [34]. The specific expression of *Chrna2* in inhibitory L5 MCs^α2^ raises questions of how important the α2 subunit is for cholinergic inputs. Several cortical interneurons express nicotinic acetylcholine receptors (nAChRs) [47–49], suggesting cholinergic modulation of inhibition in the cortex, most likely from the basal forebrain [50]. Cortical LTS cells, such as Martinotti cells [51,52], are excited by acetylcholine via nicotinic receptors and alter cortical circuit processing [53]. Cholinergic input is most likely mediated by additional nicotinic subunits that together form high affinity receptors for acetylcholine [54]. Several candidate subunits exist, but perhaps the more promising ones, judged from their specific expression in cortical L5, response to nicotine, and known co-expression with α2 subunits, include α6-nAChRs, β2-nAChRs, and β4-nAChRs [55–57]. The focus of our work has been on the functionality of Martinotti cells, not the nAChR subunits; however, earlier studies of cholinergic subunits can provide potential clues to Martinotti cell function. A deletion of α2-nAChRs has shown a normal phenotype but altered responses during nicotine-associated behaviors [58]. Deletion of α2-nAChRs has also shown reduced nicotine-induced hippocampal LTP in the temperoammonic path, most likely via oriens-lacunosum moleculare (OLM) interneurons [59,19]. Interestingly, Chrna2 is expressed in OLM cells, which target the distal dendrites of hippocampal PCs in a comparable manner as MCs^α2^ target the distal dendrites of cortical PCs. In some similarity to the suggested role for LTS cells in directing the flow of information in the cortex [53], OLM cells have been suggested to gate internal and external signals to the hippocampus [19]. In addition, MCs^α2^ might modulate cortical states, because SOM+ interneurons have recently been implied to be involved in transitions between UP and DOWN states [60]. Furthermore, studies of β2-nAChR KO mice have suggested a role for β2-containing nAChR in restricting cortical UP states and might be interesting for future studies on how nAChR are distributed in cortical interneurons such as Martinotti cells [13,61,62]. Our preparation did not examine cortical UP and DOWN states; instead, we depolarized neurons with a low concentration of the cholinergic agonist carbachol. As the IPSP amplitude generated by MCs^α2^ is dependent on the membrane potential of the postsynaptic PCs, this illustrates how MC^α2^ inhibition (amplitude of IPSPs) could alter in a state-dependent manner, thereby exerting a state-dependent modulation of PC excitability.

In summary, we report the identification of a marker specific for L5 Martinotti cells projecting to layer 1. These Martinotti cells were synaptically connected to large, thick-tufted PCs with prominent AHP and ADP, demonstrating a distinctive microcircuit between one type of interneuron and one subtype of PCs. Furthermore, we demonstrate that Martinotti cell–mediated inhibition can initiate and also maintain synchronous firing between PCs. We also show that this inhibition is frequency dependent and, when repeated in beta bursts, can continuously align firing of PCs. Lastly, using a closed-loop system in which PCs auto-synchronized their firing, we show that Martinotti cells were able to bridge the communication between unconnected PCs via activity-dependent inhibition. Thus, via their feedback and feedforward connections, Martinotti cells are important for regulating thick-tufted type A PC output in L5, most likely altering voltage-dependent dendritic properties and actively influencing somatic spike generation and synchronization.

## Materials and Methods

### Ethics Statement

All experiments were approved by the Swedish Animal Welfare authorities and followed Uppsala University guidelines for the care and usage of laboratory animals (ethics permits C132/13 and C135/14). Efforts were made to minimize the numbers of animals used.

### Mice

In this study, we used transgenic mice (both males and females), with Chrna2-Cre [19,20] that were crossed with a tdTomato fluorescence reporter line *Gt(ROSA)26Sor*^*tm14(CAG-tdTomato)Hze*^ (*R26*^*tom*^; Allen Brain Institute) or with a HaloR-expressing line Rosa26-eNphR-EYFP (HaloR; Jackson Laboratory Stock No. 014539). Cre-negative littermates were routinely used as controls.

### CLARITY

The CLARITY procedure followed a standard protocol [63]. In summary, 2–3-mo-old Chrna2-Cre/*R26*^*tom*^ mice (*n* = 2) were transcardially perfused with 20 ml of ice-cold 1x PBS solution followed by 20 ml of a hydrogel monomer solution consisting of 4% acrylamide, 0.05% bis-acrylamide, 0.25% VA-044 initiator, and 4% paraformaldehyde in PBS. Brains were quickly dissected and placed in hydrogel monomer solution for 3 d at 4°C. Prior to polymerization of the hydrogel monomer solution, samples were placed in a desiccation chamber attached to a vacuum pump. With the sample lid ajar, air was removed from the chamber for 10 min and replaced with nitrogen gas, after which the sample lid was tightly shut. The hydrogel monomer solution was polymerized by heating the samples to 37°C for 3 h in a water bath whilst shaking. Embedded tissue was extracted from the gel, and brains were sliced to 3-mm coronal sections using a brain matrix. Passive clearing of slices was achieved by repeated, 3-d washes in a 4% Sodium Dodecyl Sulphate (SDS) sodium borate buffer (200 mM, pH 8.5) solution at 45°C on a shaker plate for 6 wk. SDS was removed from the samples by incubating in PBST_0.1_ (1x PBS and 0.1% Triton X-100) on a shaker plate for two consecutive 1-d washes.

Clear tissue was refractive index-matched through serial, 1-d incubations in 20%, 40%, and 63% 2,2′-Thiodiethanol (TDE, Sigma-Aldrich) in 1x PBS solution. Light sheet fluorescence images were acquired using Zeiss light sheet Z1 with a 5x/0.16 objective. Individual image tiles were 3D-stitched using Arivis Vision4D (Arivis). Imaris 8.1 (Bitplane) was used for analysis, volume rendering, soma detection, and data visualization. Chrna2-Cre/*R26*^*tom*^ cells were counted using Imaris 8.1 (Bitplane) and Matlab (version 2013a, MathWorks) in an 800-μM section (AP: −2.40 to −3.20 mm, ML: 2.00 to 5.00 mm, and DV: 0.50 to 3.50 mm). Cells were divided into infragranular (roughly corresponding to L5/6) and supragranular (roughly corresponding to L2/3) cells by fitting a curve between ML: 4.25 mm, DV: 3.50 mm and ML: 2.00 mm, DV: 0.75 mm, roughly dorsolateral to L5.

### Immunohistochemistry

Chrna2-Cre/*R26*^*tom*^ mice (2–3 mo, *n* = 3) were anesthetized with isoflurane and decapitated before dissection. Immunohistochemistry (IHC) was performed as previously described in [19]. The following dilutions of antibodies were used: SOM antibody 1:150 (MAB354, Anti-SOM Antibody, clone YC7 [Merck Millipore Corporation]), Anti-rat Cy5, 1:500 (Invitrogen).

### Single-Cell Reverse Transcriptase PCR

Following whole-cell recordings, we used strong negative pressure to suck the cytoplasm and organelles of the cells into the recording pipette tip, similar to [19]. Buffers and cDNA conversion are further described in [19]. A two-round PCR (nested) to detect GAD1, Vglut1, or Vglut2 cDNA was done. The following mix was used for the first and second round of PCR: 1.5 mM MgCl_2_, 10 pmol of each primer, 1.0 U of platinum *Taq*-DNA polymerase (Invitrogen), 20 mM Tris·HCl, and 50 mM KCl pH 8.4; thermal cycle: 94°C/2-min denaturation step followed by 35 cycles of 94°C/50 s, 55°C/45 s, and 72°C/45 s. In the second round, instead of mixing the original template, 10% of the first PCR reaction as template was used. Second-round PCR products were visualized on 2% agarose gels. Primers were designed based upon sequences deposited in the GenBank database (www.ncbi.nlm.nih.gov/nucleotide). The primers used were as follows: first round: GAD1: CCAATAGCCTGGAAGAGAAGAG (*forward*), TCCCATCACCATCTTTATTTGA (*reverse*); Vglut1: CGCTACATCATCGCCATCATGAG (*forward*), GGAGGGGCCCATTTGCTCCA (*reverse*); Vglut2: GCCGCTACATCATAGCCATC (*forward*), GCTCTCTCCAATGCTCTCCTC (*reverse*); second round: GAD1: CCAATAGCCTGGAAGAGAAGAG (*forward*), TCCCATCACCATCTTTATTTGA (*reverse*); Vglut1: CTGGAGGATTTATCTGCCAAAAAT (*forward*), GGTATGTGACCCCCTCCACCAAT (*reverse*); Vglut2: ACATGGTCAACAACAGCACTATC (*forward*), ATAAGACACCAGAAGCCAGAACA (*reverse*).

### Electrophysiology

Coronal slices from Chrna2-Cre/*R26*^*tom*^ transgenic mice (P19–29, *n* = 12) were obtained similar to [24]. In summary, brains were rapidly removed and placed in ice-cold sucrose/artificial cerebrospinal fluid (ACSF) consisting of the following (in mM): KCl, 2.49; NaH2PO4, 1.43; NaHCO3, 26; glucose, 10; sucrose, 252; CaCl2, 1; MgCl2, 4. Coronal 300-μm-thick slices containing the primary auditory cortex were cut using a vibratome (VT1200, Leica, Microsystems) and were subsequently moved to a submerged holding chamber containing normal ACSF (in mM): NaCl, 124; KCl, 3.5; NaH2PO4, 1.25; MgCl2, 1.5; CaCl2, 1.5; NaHCO3, 30; glucose,10, constantly bubbled with 95% O_2_ and 5% CO_2_ and kept at 35°C for 1 h then maintained at room temperature. The slices were transferred to submerged chamber under an upright microscope equipped with DIC optics (Olympus) and perfused with oxygenated ASCF (1–1.25 ml/min) at 30°C. For some experiments, carbachol (10 μM, Sigma-Aldrich) and ZD7288 (20 μM, Tocris Cookson Inc.) were added to the perfusate. Patch pipettes from borosilicate glass capillaries (GC150F-10 Harvard Apparatus) were pulled on a vertical puller (Narishige, Japan) with resistance around 7 MΩ. Pipettes were filled with internal solution containing (in mM) the following: K-gluconate, 130; NaCl, 7; MgCl2, 2; ATP, 2; GTP, 0.5; HEPES, 10; EGTA, 0.1 (pH was adjusted to 7.2 using KOH). Whole-cell current clamp recordings were acquired using a Multiclamp 700B amplifier (Axon Instruments, CA, USA) and digitized with a Digidata 1440A data acquisition card (Axon Instruments, CA, USA). WinWCP and WinEDR softwares implemented by Dr. J. Dempster (University of Strathclyde, Glasgow, UK) were used to record electrophysiological signals.

Patch-clamp data were analyzed with custom routines in MATLAB. APs were triggered by 500-ms depolarizing current injections from 10–100 pA. The first fired AP in response to minimal current injection was analyzed for AP amplitude (peak to AHP voltage), threshold (where the change in membrane potential exceeds 20 mV/ms), half-width (halfway between threshold voltage and peak), and first spike latency (time between stimulus onset and the AP threshold of the first spike). AHPs were analyzed for magnitude (AP threshold—minimum of voltage trough between the first and the second AP in a spike train). Spike rate was calculated as the number of APs per 1,000 ms. Spike-frequency adaptation was measured as the inverse of the mean of the last three interspike intervals (steady-state frequency) divided by the inverse of the first interspike interval (maximum frequency) in response to 100-pA current injections and subtracted from 100% (no adaptation). In spike-frequency adaptation plots, the reciprocal of consecutive interspike intervals is shown for each AP versus the time after onset of the current pulse.

ChR2 stimulation frequencies (2, 5, 15, 25, 40, and 70 Hz) and HaloR-evoked hyperpolarization (5, 10, 100, 250, 500, and 1000 ms) were applied in randomized order to avoid statistical dependencies between cases. ChR2-triggered IPSPs and FDDI were measured as amplitude, time to peak, and half decay time, for which the onset was defined as the time at which the potential exceeded three times the standard deviation of the preceding baseline. HaloR-evoked hyperpolarization amplitudes were quantified as the difference between resting membrane potential and the peak of hyperpolarization. Rebound APs were quantified by number, maximum frequency, and duration (time of last minus time of first rebound AP). Burst-spiking PCs were distinguished from single-spiking PCs by obtaining the interspike intervals, at which burst-spiking PCs showed an increased amount of short (≤20 ms) interspike intervals at near-threshold potentials.

### Imaging and Tracing

MCs^α2^ were identified by cortical tdTomato-expression in Chrna2-Cre/*R26*^*tom*^ mice that were perfused as previously described [19], and 20-μm- and 60-μm-thick coronal slices were imaged using a Zeiss LSM 510 Meta confocal microscope. Cells targeted by electrophysiology experiments were routinely filled with biocytin and stained with streptavidin-488 nm for post-hoc analysis. Images were collected on a Zeiss LSM 510 Meta confocal microscope and stacked and 2D-stichted using ImageJ 1.50a (NIH), where the color palette was adjusted for consistency (tdTomato—*red*, biocytin/ChR2—*green*). Soma detection and Neurite tracings were done semi-automated with NeuronJ 1.4.2 (ImageJ Plugin) or fully automated with Imaris 8.1 FilamentTracer (Bitplane) using “Autopath” in the algorithm settings and the threshold mechanism to correct for over-/under-sampled tracings following the image intensity.

### Optogenetics

Chrna2-Cre/*R26*^*tom*^ mice (1–2 mo old, *n* = 33) were anesthetized with Isofluran (1%–4%) and placed on a heat pad, with the head fixed with a nose holder and ear bars in a stereotaxic frame (Stoelting Co.). The skin was cleaned with iodine and opened with a straight incision, and the bregma was identified using small amount of peroxide. The coordinates for bilateral virus injection were as follows: AP: −2.46 mm, ML: +/−4.00 mm, and DV: 2.00/2.50 mm. We used bilateral injections to obtain the maximum number of slices containing the primary auditory cortex, with preserved dendritic trees of the type A PCs in layer 1 (on average two slices, 300 μm thick, per hemisphere) per animal. A small hole was drilled in the skull using a dental micro drill, causing minimal bleeding during the process. Viral vectors (pAAV-EF1a-double floxed-hChR2(H134R)-EYFP-WPRE-HGHpA, University of Pennsylvania Vector Core Facility) in solution (6.2 x10^12^ / 1.6×10^13^ particles/ml) of 0.50–1.00 μl were slowly infused (0.10 μl/min) into the auditory cortex at two depths (2.00 and 2.50 mm) using a Hamilton 10-μl syringe and an infusion/withdraw pump (World Precision Instruments/KD Scientific). After infusion, the needle was left in place for 1–5 min to allow complete diffusion of the virus. Next, the scalp was rehydrated with saline and sutured with 4–5 stitches and local anesthesia (a drop of Lidocaine/Marcaine) applied onto the sutured skin before the mouse was allowed to wake up. The animal was monitored and kept warm until fully awake (moving and starting to eat and drink water). Mice were killed after approximately 3–6 wk for in vitro electrophysiological experiments and/or histological procedures.

### Optical Feedback Inhibition

We modified our dynamic clamp system [30] running the Real Time Application Interface for Linux-based (RTAI) from the Politecnico di Milano Institute-Dipartimento di Ingegneria Aerospaziale (Mantegazza, http://www.rtai.org/) on a Dell Precision T1500 with a Quad-Core Intel Core i7 with 2.80 Ghz, 5.8 Gigabyte memory, and a National Instruments DAQ card (NI PCI-6251). Routines for data acquisition were programmed in GNU-C using the Linux Control and Measurement Device Interface (COMEDI). The membrane potentials of the patched cells were acquired in 20 kHz, and APs were detected based on threshold (>−20 mV) in real time, triggering the LED (CoolLED pE-1) via 3-ms (ChR2 experiments) or 500-ms (HaloR experiments) TTL pulses.

### Data Analysis

Matlab (version 2013a, MathWorks) was used for data analysis. APs from MCs^α2^ and PCs were detected based on threshold (>−20 mV). PC spike trains were transformed into a series of 0 (no spike) and 1 (spike), with 0.1-ms precision (binning). Accordingly, kernel density estimates are probability densities and were computed on population data (all patched PCs) with 0.1 “bandwidth” (“ksdensity” command in Matlab). The kernel density estimates show the distribution of APs over time and highlight increased (peaks) and decreased (valleys) co-occurrences of spikes. Power spectral density analysis of the binary spike series was made using Welch’s method (“pwelch” command in Matlab) to find the frequency components with highest power. Coherence was calculated pairwise (i.e., for simultaneously patched PCs, the similarity between the binary sequences of the two PCs was calculated) and plotted as mean over all recordings (“cohere” command in Matlab) to investigate the dependence of two cells as a function of frequency. Cross-correlograms were calculated using the “coeff” option of the cross-correlation command in Matlab (to scale the cross-correlation values from −1 to 1 and prevent dependency of the cross-correlation on the number of spikes) and then smoothed by a moving average filter with a span of 10 ms to find the functional dependence between the APs of simultaneously recorded cells over time. We displayed cross-correlations over a lag range of ±1 s. The synchrony index was defined as the maximum peak of the normalized cross-correlograms between −50 ms and 50 ms, as previously described [30], with 0 ≙ no synchronization and 1 ≙ full synchronization. To investigate statistical dependence, mutual information MI(PCleft, PCright) was determined on the binary spike data (binning = 20 ms) by calculating the distributions of the spike trains (univariate distribution of each spike train separately as well as bivariate distribution of both spike trains) and expressing them as entropies H(PCleft) and H(PCright), meaning how “diverse” the spike trains were. The sum of the two entropies H(PCleft) and H(PCright) minus the joint entropy H(PCleft, PCright) quantified the conditional entropy, i.e., the mutual information MI(PCleft, PCright). The MI was formulated as a mutual information index with a scaling factor of 1,000 [30].

### Statistical Analysis

All statistical analysis was performed using R version 3.2.3 (Foundation for Statistical Computing, Vienna, Austria). Data are reported as mean ± standard error of the mean (SEM) and plotted as bar plots or box plots. Data larger than q3 + 1.5*(q3–q1) or smaller than q1–1.5*(q3–q1), with q1 and q3 denoting the 25th and 75th percentiles (see box plots), were considered as outlier and discarded. Statistical comparisons were determined using two-tailed Student’s paired *t* test, and to account for multiple comparisons, the data were analyzed using ANOVA and post-hoc test with Tukey correction (* ≙ *p* < 0.05, ** ≙ *p* < 0.01, *** ≙ *p* < 0.001, and **** ≙ *p* < 0.0001). The order of stimulations of different frequencies (e.g. 2, 5, 15, 25, 40, 70 Hz) was systematically varied to avoid statistical dependencies between the timing of recordings and the frequency investigated.

## Supporting Information

S1 Fig Tomato+ cells of Chrna2-Cre/*R26*^*tom*^ mice across cortical areas. (A) *Left*; 4x coronal images (60 μm thick) of primary auditory cortex and (and parts of secondary visual cortex—left image; *top*). Cell bodies of tdTomato+ neurons (red) appear in layer 5 and dense axonal arborizations are shown in layer 1 (image at approx. bregma -2.46 mm). The *star* highlights the oriens layer of the hippocampus and *arrowhead* shows dense axonal projections of oriens lacunosum-moleculare cells [19]. *Right*; 10x magnification of square area outlined in *left*. (B) Coronal slices (4x (*left*) and 10x (*right*) magnification) of the medial prefrontal cortex where the corpus callosum was not yet joined (around bregma +1.78 mm). (C) Parasagittal slices (10° angle), approximately 1.92 mm lateral to the midline (4x (*left*) and 10x (*right*) magnification), showing distribution of tdTomato+ cell bodies (red) in the primary somatosensory cortex, primary motor cortex and secondary visual cortex. Red squares show the approximate location of the 4x image (inset), white dashed squares for the 10x images. Note the dense axonal ramifications of Chrna2-Cre/*R26*^*tom*^ cells in layer 1 (star). Scale bars = 400 μm (*left*) and 200 μm (*right*) resp. (D) Immunohistochemistry for somatostatin (left) in a cortical section from a Chrna2-Cre/*R26*^*tom*^ mouse (middle, *star* highlighting the dense axonal ramifications of Chrna2-Cre/*R26*^*tom*^ cells in layer 1) to visualize co-expression with chrna2 (right, arrowheads). Scale bar = 100 μm. (E) A total 792 cells were counted; 297 cells were Chrna2+, 495 were somatostatin+ and 90 of these were double labelled for both Chrna2 and somatostatin (n = 3 mice, 8 sections of 35 μm thickness). Venn diagrams for all layers (layer1-6) and layer 5 visualize the overlap (somatostatin -grey; chrna2 –red and co-expression -pink). Insets in top corner of all panels show mouse brain atlas schematics of area show. (F) Electrophoresis gel image from the single cell analysis showing 6 positive cells (columns 2–4 and 6–8) for GAD1+, 1 negative cell (column 5) and the negative control (column 1).
(TIF)

S2 Fig Chrna2-Cre/*R26*^*tom*^ cells show typical Martinotti cell morphology and putative interconnections with PCs. (A) Example of biocytin-filled (green) Chrna2-Cre/*R26*^*tom*^ cell highlighting the long axonal projection to layer 1 (*left*, à) emerging from the main dendrite (*right*, circle). Note thick main trunks of dendrites of other red Chrna2-Cre/*R26*^*tom*^ cell in the vicinity also pointing in the direction of layer 1. (B) Overview of the long axonal projection (à) of a biocytin filled (green) Chrna2-Cre/*R26*^*tom*^ cell, showing proximal axonal arborizations (à) with main axons extending to layer 1. Note the dense axonal ramifications in layer 1 (star). (C) i) High magnification image (63x) of layer 1 (showing biocytin-filled (green) projections from one filled thick-tufted PC and a MC^α2^ cell, also green-yellow. The thin green-yellow MC^α2^ axon (highlighted with à) could be followed visually and the high magnification image shows that it passes in close proximity to the thick dendrite of the PC, which was shown to be synaptically coupled with the recorded MC^α2^. The image is a collapsed z-stack composed of 40 (1 μm sections). ii) Close-up of the image in (i) but only showing collapsed z-stack of 10 images, to give a higher resolution, and still provide a pseudo 3D image of putative connections between the thin axon of the MC^α2^ and the thick dendrite of the PC. iii) Image showing the corresponding cell bodies of the PC and MC^α2^ (yellow) in the images on the left. Note also putative connections (arrow) from the PC to the red (not patched) chrna2-Cre/*R26*^*tom*^ cell in the lower part of the image. Scale bars = 20 μm.
(TIF)

S3 Fig MCs^α2^ are consistently activated by short duration blue light pulses and accommodating during continuous blue light stimulation. (A). Comparison of evoked IPSPs in type A PCs following (*left*) action potentials generated by brief current injection (50 pA, 3 ms) in a connected MC^α2^, and (*right*) brief light stimulation (488 nm, 3 ms) of a population of ChR2+ MC^α2^. Graphs show comparison between amplitude, time to peak and half decay time of electrically (white) and optogenetically (blue) evoked IPSPs (n = 12 cells, n = 54 IPSPs with outliers removed, see methods). Values are shown in S2 Data. All comparisons: *** ≙ p < 0.001, mean ± SEM, two-tailed Student’s paired t-test. (B) Continuous blue light stimulation (500 ms) generates adaptation in MC^α2^ firing. (C) Continuous blue light of 1000 ms fails to generate prolonged firing in ChR2-expressing MCs^α2^. (D) Increasing light intensities (500 ms continuous blue light between 0.5 and 6 mW) does not improve spike capability of ChR2-expressing MCs^α2^.
(TIF)

S4 Fig Properties of HaloR+ MCs^α2^ rebound spikes. (A) Rebound APs of HaloR+ MCs^α2^ following different durations (100, 250, 500 and 1000 ms) of continuous green light (12 repetitions) in the presence of carbachol (V_m_ = -48 mV). Note that 100 ms of green light fails to consistently evoke rebound APs (failures highlighted by arrow), while 500ms light was sufficient to generate a burst of rebound spikes. (B) Bar graphs show quantifications of number of rebound spikes, the peak hyperpolarization amplitude, rebound maximum frequency, rebound duration and time to peak of hyperpolarization. A 500 ms light stimulation was necessary to generate a burst of rebound spikes (>1), the hyperpolarization amplitude (during light) reached a plateau of -26.66 ± 0.17 mV (*top right*) (n = 12 cells, mean ± SEM, ANOVA). 1000 ms light does not increase maximum frequency of rebound APs but increases the rebound duration (*bottom left*) (n = 12 cells, mean ± SEM, two-tailed Student’s paired t-test). Quantification of hyperpolarization time to peak shows that there is no difference ≥ 250ms of light stimulation. All comparisons: * ≙ p < 0.05, ** ≙ p < 0.01, *** ≙ p < 0.001 and **** ≙ p < 0.0001. (C) MC^α2^ rebound APs generated by either negative current steps (0–100 pA) or by 500 ms green light are both resistant to ZD7288 (20 μM), a blocker of the hyperpolarization-activated cation current (I_h_).
(TIF)

S5 Fig Comparison of depolarization induced and carbachol induced firing of type A PCs and MCs^α2^. Power spectral density plots (95% confidence interval in grey, mean in black) of type A PCs (*top*) and MCs^α2^ (*bottom*) are shown in response to a continuous (+40 pA) current injection (*left*) and following (*right*) carbachol (10 μM) bath application. Continuously adding carbachol to the perfusate increased the spontaneous firing frequency of both cells, with a broad peak (therefore not at any specific frequency) in the power spectrum at 4.90 Hz (range: 0.62 to 9.39 Hz, n = 60 cells) for type A PCs and 16.02 Hz (range: 9.16 to 23.65 Hz, n = 24 cells) for MCs^α2^.
(TIF)

S6 Fig Characteristics of compound IPSPs generated by a burst of APs of HaloR-expressing MCs^α2^. (A) *Top*; Schematic of circuit. Green light stimulation (500 or 1000 ms) hyperpolarizes HaloR-expressing MCs^α2^ and upon termination of light the MCs^α2^ fired a burst of rebound APs. The corresponding compound IPSP in type A PCs represent the response to the first 3–4 MCs^α2^ spikes. Grey dashed lines highlight nicks in the trace where individual IPSPs are summed. (B) Example traces of type A PC IPSPs (grey traces, mean response in black) evoked by rebound APs following silencing a population of HaloR-expressing MCs^α2^ by green light of different duration (*left*: 500 ms; *right*: 1000 ms) for burst-spiking and (*top*) single-spiking (*bottom*) type A PCs. (C) IPSP amplitudes (*left*), time to peak (*middle*) and half decay time (*right*) in single-spiking and burst-spiking type A PCs vary depending on the light-duration (i.e. time of MC^α2^ inhibition and the subsequent rebound APs; n = 12 IPSPs, mean ± SEM, ANOVA). All comparisons: * ≙ p < 0.05, ** ≙ p < 0.01, *** ≙ p < 0.001 and **** ≙ p < 0.0001. Values are shown in S6 Data.
(TIF)

S7 Fig Stimulation frequencies for MCs^α2^ that do not lead to synchronization of type A PCs. Population response of 12 dual recordings of unconnected type A PCs (left, n = 24 cells; 12 black and 12 grey PC spike trains) during different frequencies of pulsed light stimulation (5 Hz, 25 Hz, 40 Hz and 70 Hz) of ChR2-expressing MCs^α2^ and without light stimulation (second half of spike train), to compare if MC activity could synchronize PC firing. Kernel density estimates (orange trace) show increased (peaks) and decreased (valleys) co-occurances of APs. Mean power spectral density plots for each tested frequency (right) revealed no particular peak that indicated increased synchronization for 5, 25, 40 or 70 Hz.
(TIF)

S8 Fig Firing pattern of depolarized layer 5 PCs change from bursting to non-bursting upon inhibition by MCs^α2^ at 15 Hz. (A) *Inset*: Experimental set up and indication of how the PC firing pattern is altered/not altered by 15Hz MCs^α2^ activity. Voltage traces of a single-spiking (*top*) and a burst-spiking (*bottom*) type A PC at V_m_ = -48 mV (bath application of carbachol). Frames highlight typical APs. *Bottom orange box*; Note the change from doublet-spiking to single-spiking shortly after the initiation of MCs^α2^ inhibition at 15 Hz (by activating ChR2-expressing MCs^α2^ as indicated by blue dots above traces). (B) Voltage traces in (A) but at V_m_ = -53 mV shows that the change of firing pattern only happens at depolarized potentials, probably due to the stronger inhibition (larger Cl^-^ drive at depolarized potentials). Frames highlight examples of APs at a zoomed in timescale.
(TIF)

S1 Data Data for Fig 1G and 1H. (XLSX)

S2 Data Data for S3A Fig. (XLSX)

S3 Data Data for Fig 3D and 3E. (XLSX)

S4 Data Data for Fig 4A, 4B, 4D and 4E. (XLSX)

S5 Data Data for S4B Fig. (XLSX)

S6 Data Data for S6C Fig. (XLSX)

S7 Data Data for Fig 5A and 5B. (XLSX)

S8 Data Data for Fig 6C. (XLSX)

S9 Data Data for Fig 7B. (XLSX)

S1 Movie Tomato+ cells of Chrna2-Cre/*R26*^*tom*^ mice visualized across cortical areas. A series of images from adult (2 months old) Chrna2-Cre/*R26*^*tom*^ mouse cortex (coronal slice, 1300 μm thickness) after CLARITY processing is shown. Please note the second band of tomato+ cells highlighted in the stratum oriens of hippocampus [19] and the dense axonal arborisation in stratum lacunosum-moleculare, highlighted as a grey dense mass.
(MP4)

S1 Text Supporting Information. (DOCX)

## Abbreviations

ADP: afterdepolarization
AHP: afterhyperpolarization
AP: action potential
ChR2: Channelrhodopsin-2
Chrna2: nicotinic acetylcholine receptor α2 subunit
Cre: cyclization recombinase
EPSC: excitatory postsynaptic current
EPSP: excitatory postsynaptic potential
FDDI: frequency-dependent disynaptic inhibition
GAD1: Glutamate decarboxylase 1
GIN cell: a type of somatostatin-expressing interneuron defined by its green fluorescent protein expression in a transgenic mouse
HaloR: Halorhodopsin
IPSP: inhibitory postsynaptic potential
L5: layer 5
LTS: low-threshold spiking
MCs^α2^: L5-specific Chrna2-Martinotti cells
nAChR: nicotinic acetylcholine receptor
OLM: oriens-lacunosum moleculare
PC: pyramidal cell
RT-PCR: reverse transcription PCR
SEM: standard error of the mean
SOM: somatostatin
VIP: vasoactive intestinal peptide
