# Supplementary figures and images for: Chrna2-Martinotti Cells Synchronize Layer 5 Type A Pyramidal Cells via Rebound Excitation
Source: PLoS Biol. 2017 Feb 9;15(2):e2001392. doi: 10.1371/journal.pbio.2001392 (PMC5300109; doi:10.1371/journal.pbio.2001392)

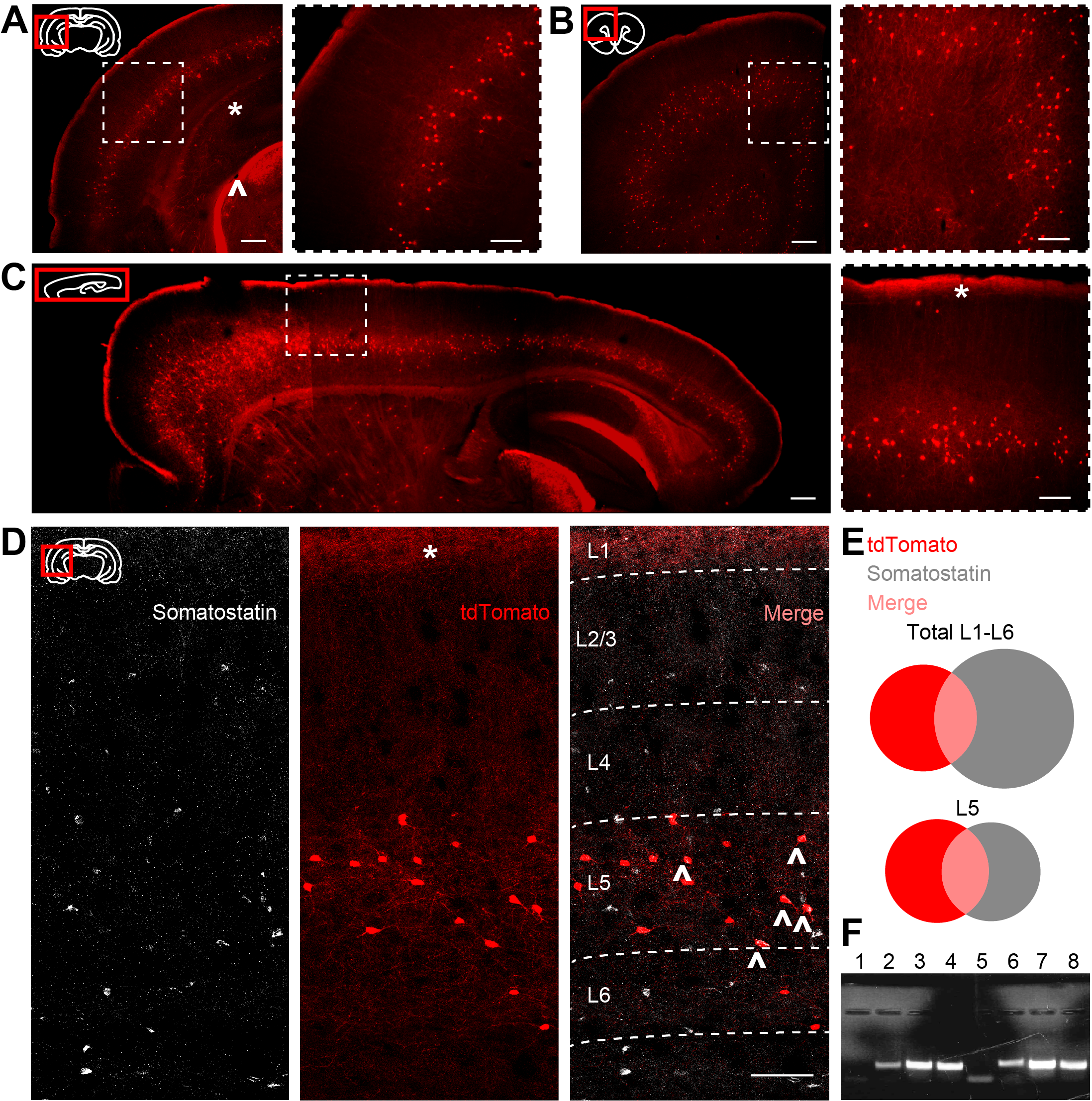

Supplement: S1 Fig — (A) Left; 4x coronal images (60 μm thick) of primary auditory cortex and (and parts of secondary visual cortex—left image; top). Cell bodies of tdTomato+ neurons (red) appear in layer 5 and dense axonal arborizations are shown in layer 1 (image at approx. bregma -2.46 mm). The star highlights the oriens layer of the hippocampus and arrowhead shows dense axonal projections of oriens lacunosum-moleculare cells [19]. Right; 10x magnification of square area outlined in left. (B) Coronal slices (4x (left) and 10x (right) magnification) of the medial prefrontal cortex where the corpus callosum was not yet joined (around bregma +1.78 mm). (C) Parasagittal slices (10° angle), approximately 1.92 mm lateral to the midline (4x (left) and 10x (right) magnification), showing distribution of tdTomato+ cell bodies (red) in the primary somatosensory cortex, primary motor cortex and secondary visual cortex. Red squares show the approximate location of the 4x image (inset), white dashed squares for the 10x images. Note the dense axonal ramifications of Chrna2-Cre/R26tom cells in layer 1 (star). Scale bars = 400 μm (left) and 200 μm (right) resp. (D) Immunohistochemistry for somatostatin (left) in a cortical section from a Chrna2-Cre/R26tom mouse (middle, star highlighting the dense axonal ramifications of Chrna2-Cre/R26tom cells in layer 1) to visualize co-expression with chrna2 (right, arrowheads). Scale bar = 100 μm. (E) A total 792 cells were counted; 297 cells were Chrna2+, 495 were somatostatin+ and 90 of these were double labelled for both Chrna2 and somatostatin (n = 3 mice, 8 sections of 35 μm thickness). Venn diagrams for all layers (layer1-6) and layer 5 visualize the overlap (somatostatin -grey; chrna2 –red and co-expression -pink). Insets in top corner of all panels show mouse brain atlas schematics of area show. (F) Electrophoresis gel image from the single cell analysis showing 6 positive cells (columns 2–4 and 6–8) for GAD1+, 1 negative cell (column 5) and the negative [file pbio.2001392.s001.tif]

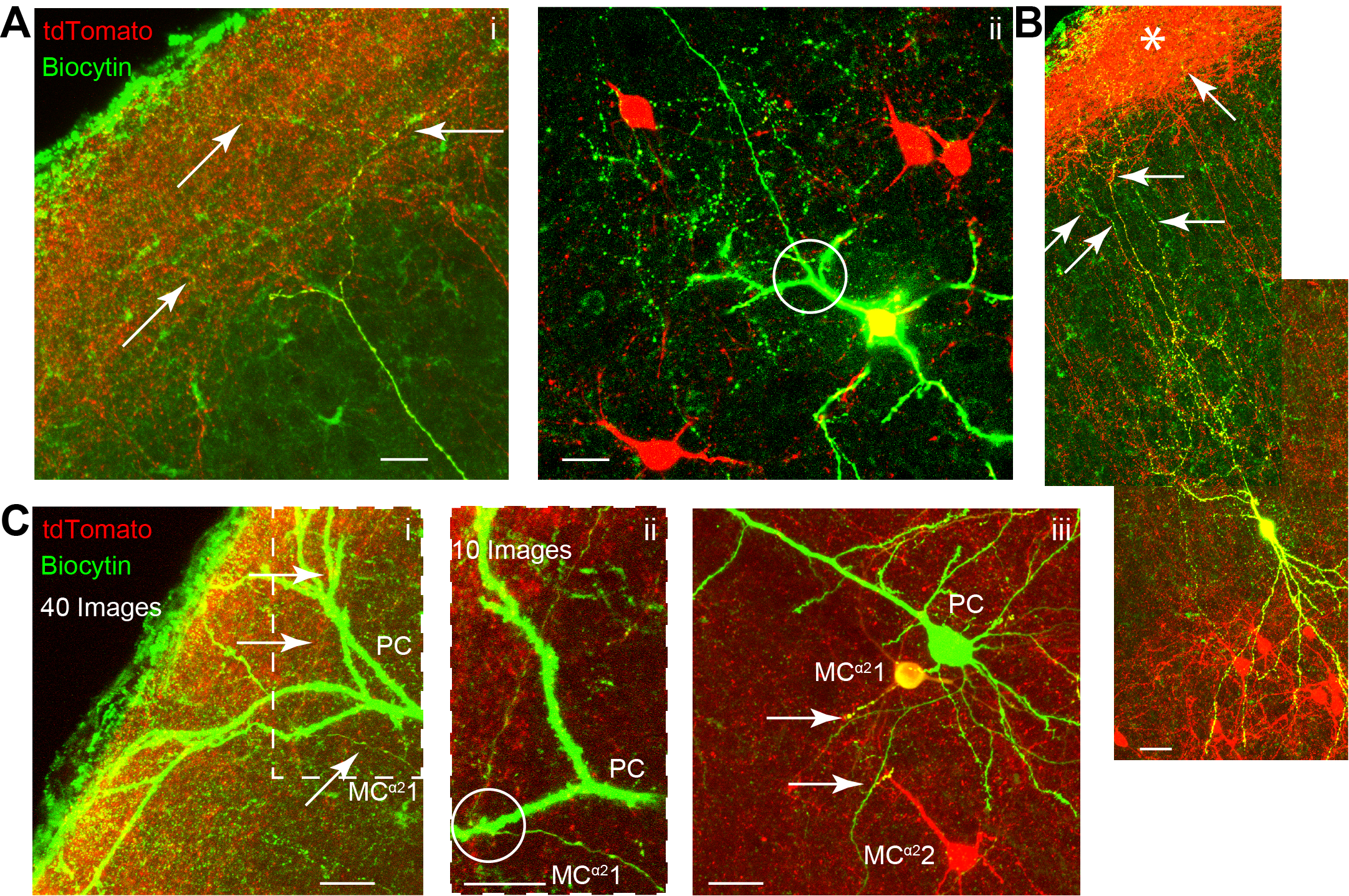

Supplement: S2 Fig — (A) Example of biocytin-filled (green) Chrna2-Cre/R26tom cell highlighting the long axonal projection to layer 1 (left, à) emerging from the main dendrite (right, circle). Note thick main trunks of dendrites of other red Chrna2-Cre/R26tom cell in the vicinity also pointing in the direction of layer 1. (B) Overview of the long axonal projection (à) of a biocytin filled (green) Chrna2-Cre/R26tom cell, showing proximal axonal arborizations (à) with main axons extending to layer 1. Note the dense axonal ramifications in layer 1 (star). (C) i) High magnification image (63x) of layer 1 (showing biocytin-filled (green) projections from one filled thick-tufted PC and a MCα2 cell, also green-yellow. The thin green-yellow MCα2 axon (highlighted with à) could be followed visually and the high magnification image shows that it passes in close proximity to the thick dendrite of the PC, which was shown to be synaptically coupled with the recorded MCα2. The image is a collapsed z-stack composed of 40 (1 μm sections). ii) Close-up of the image in (i) but only showing collapsed z-stack of 10 images, to give a higher resolution, and still provide a pseudo 3D image of putative connections between the thin axon of the MCα2 and the thick dendrite of the PC. iii) Image showing the corresponding cell bodies of the PC and MCα2 (yellow) in the images on the left. Note also putative connections (arrow) from the PC to the red (not patched) chrna2-Cre/R26tom cell in the lower part of the image. Scale bars = 20 μm. (TIF) [file pbio.2001392.s002.tif]

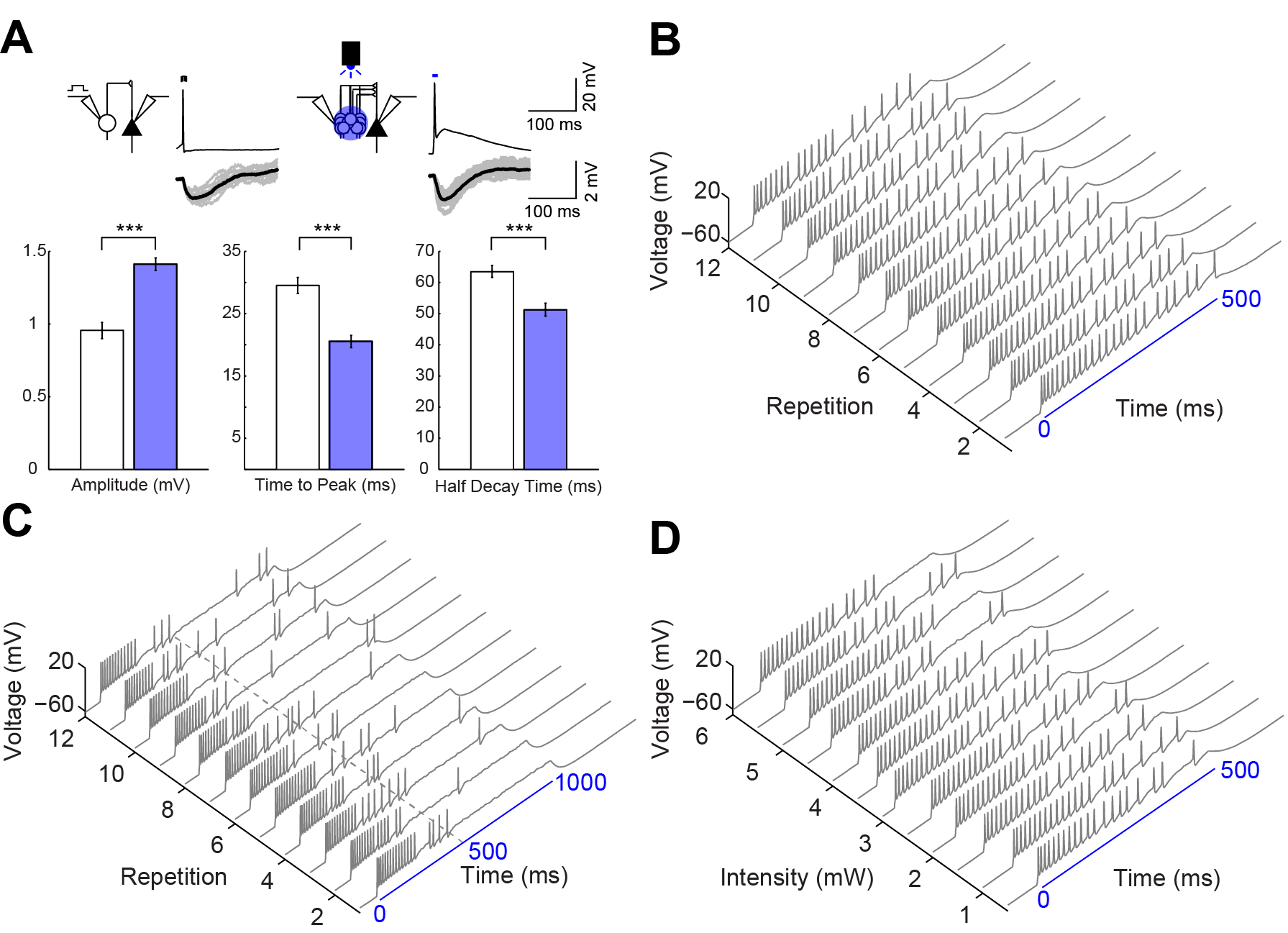

Supplement: S3 Fig — (A). Comparison of evoked IPSPs in type A PCs following (left) action potentials generated by brief current injection (50 pA, 3 ms) in a connected MCα2, and (right) brief light stimulation (488 nm, 3 ms) of a population of ChR2+ MCα2. Graphs show comparison between amplitude, time to peak and half decay time of electrically (white) and optogenetically (blue) evoked IPSPs (n = 12 cells, n = 54 IPSPs with outliers removed, see methods). Values are shown in S2 Data. All comparisons: *** ≙ p < 0.001, mean ± SEM, two-tailed Student’s paired t-test. (B) Continuous blue light stimulation (500 ms) generates adaptation in MCα2 firing. (C) Continuous blue light of 1000 ms fails to generate prolonged firing in ChR2-expressing MCsα2. (D) Increasing light intensities (500 ms continuous blue light between 0.5 and 6 mW) does not improve spike capability of ChR2-expressing MCsα2. (TIF) [file pbio.2001392.s003.tif]

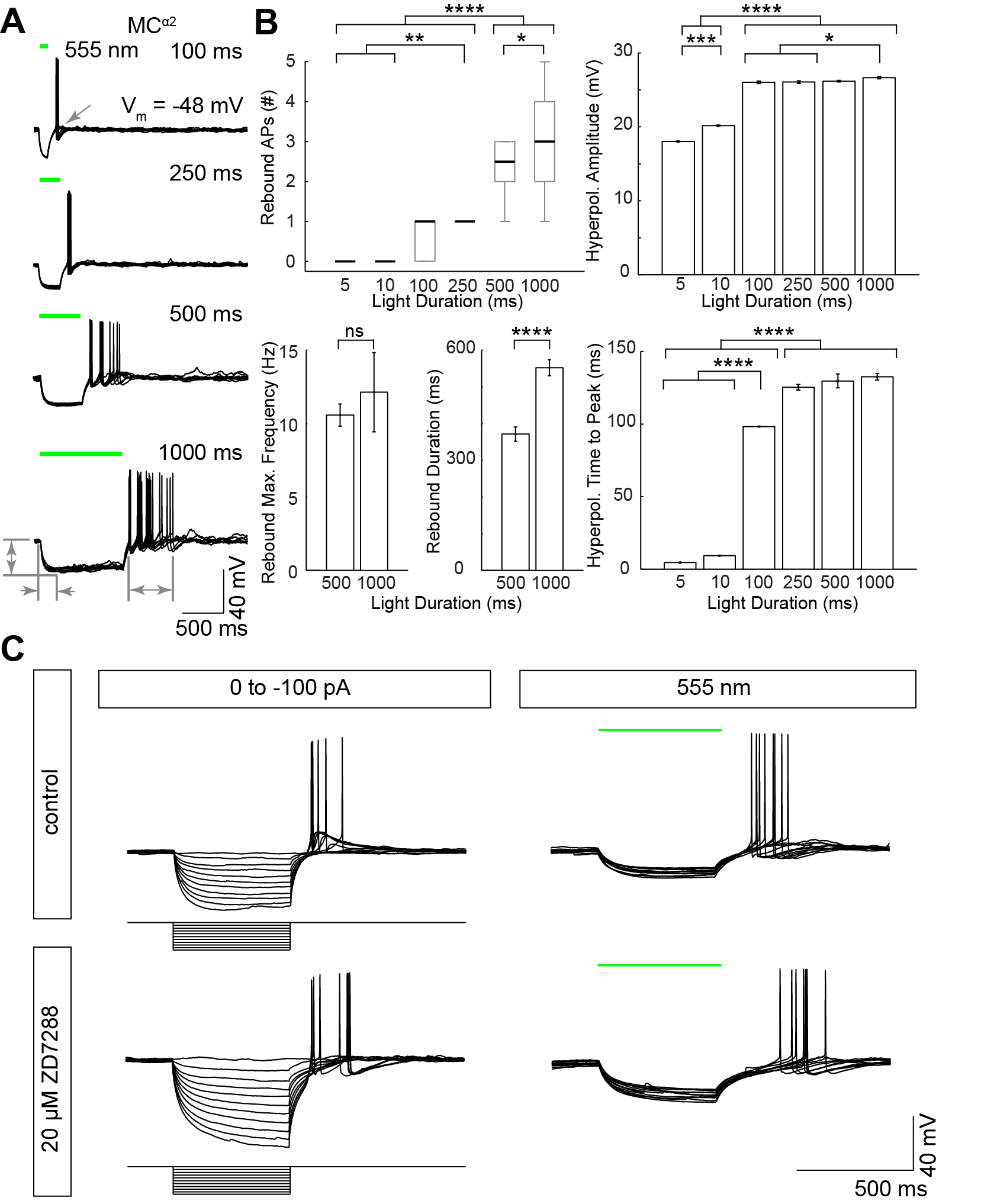

Supplement: S4 Fig — (A) Rebound APs of HaloR+ MCsα2 following different durations (100, 250, 500 and 1000 ms) of continuous green light (12 repetitions) in the presence of carbachol (Vm = -48 mV). Note that 100 ms of green light fails to consistently evoke rebound APs (failures highlighted by arrow), while 500ms light was sufficient to generate a burst of rebound spikes. (B) Bar graphs show quantifications of number of rebound spikes, the peak hyperpolarization amplitude, rebound maximum frequency, rebound duration and time to peak of hyperpolarization. A 500 ms light stimulation was necessary to generate a burst of rebound spikes (>1), the hyperpolarization amplitude (during light) reached a plateau of -26.66 ± 0.17 mV (top right) (n = 12 cells, mean ± SEM, ANOVA). 1000 ms light does not increase maximum frequency of rebound APs but increases the rebound duration (bottom left) (n = 12 cells, mean ± SEM, two-tailed Student’s paired t-test). Quantification of hyperpolarization time to peak shows that there is no difference ≥ 250ms of light stimulation. All comparisons: * ≙ p < 0.05, ** ≙ p < 0.01, *** ≙ p < 0.001 and **** ≙ p < 0.0001. (C) MCα2 rebound APs generated by either negative current steps (0–100 pA) or by 500 ms green light are both resistant to ZD7288 (20 μM), a blocker of the hyperpolarization-activated cation current (Ih). (TIF) [file pbio.2001392.s004.tif]

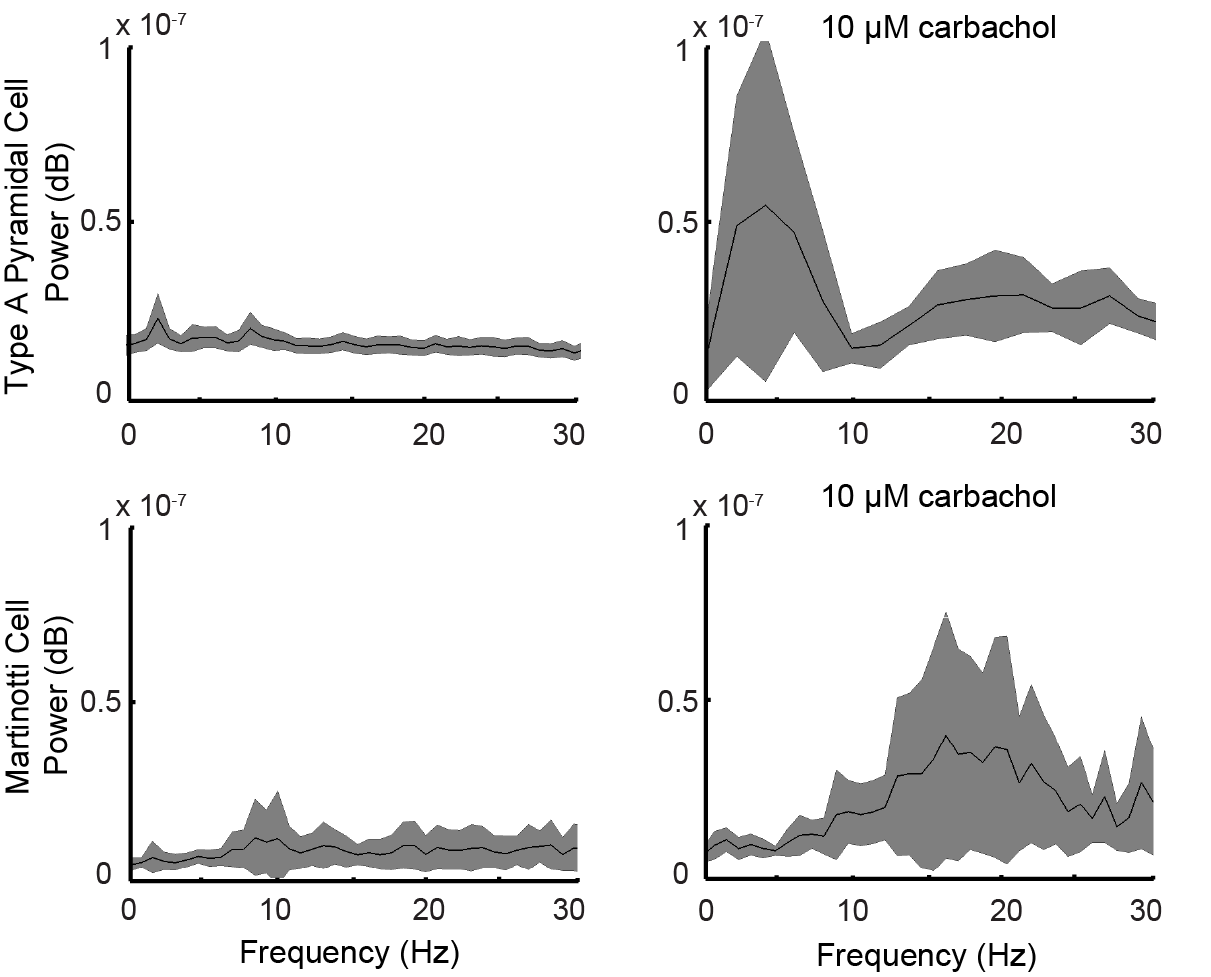

Supplement: S5 Fig — Power spectral density plots (95% confidence interval in grey, mean in black) of type A PCs (top) and MCsα2 (bottom) are shown in response to a continuous (+40 pA) current injection (left) and following (right) carbachol (10 μM) bath application. Continuously adding carbachol to the perfusate increased the spontaneous firing frequency of both cells, with a broad peak (therefore not at any specific frequency) in the power spectrum at 4.90 Hz (range: 0.62 to 9.39 Hz, n = 60 cells) for type A PCs and 16.02 Hz (range: 9.16 to 23.65 Hz, n = 24 cells) for MCsα2. (TIF) [file pbio.2001392.s005.tif]

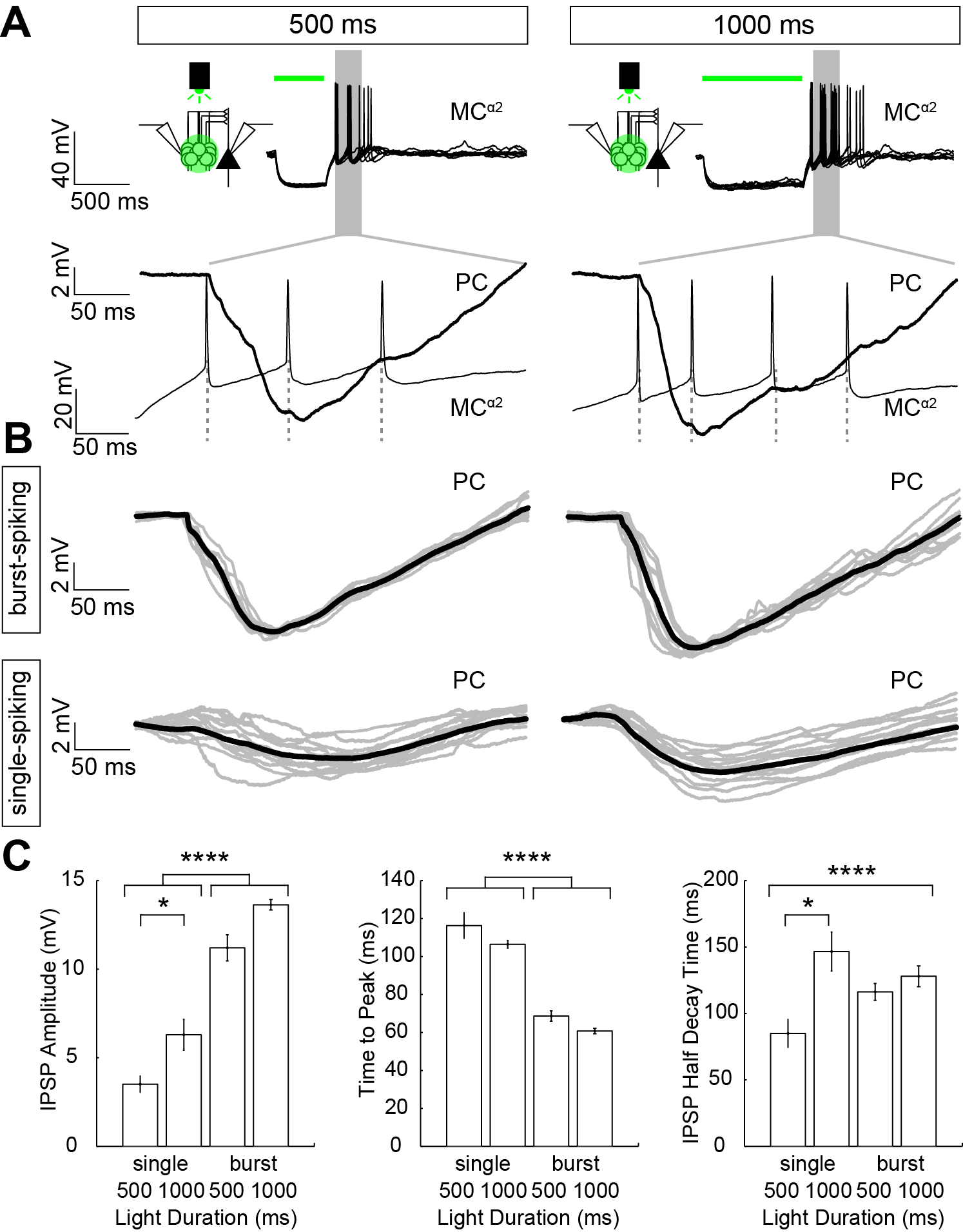

Supplement: S6 Fig — (A) Top; Schematic of circuit. Green light stimulation (500 or 1000 ms) hyperpolarizes HaloR-expressing MCsα2 and upon termination of light the MCsα2 fired a burst of rebound APs. The corresponding compound IPSP in type A PCs represent the response to the first 3–4 MCsα2 spikes. Grey dashed lines highlight nicks in the trace where individual IPSPs are summed. (B) Example traces of type A PC IPSPs (grey traces, mean response in black) evoked by rebound APs following silencing a population of HaloR-expressing MCsα2 by green light of different duration (left: 500 ms; right: 1000 ms) for burst-spiking and (top) single-spiking (bottom) type A PCs. (C) IPSP amplitudes (left), time to peak (middle) and half decay time (right) in single-spiking and burst-spiking type A PCs vary depending on the light-duration (i.e. time of MCα2 inhibition and the subsequent rebound APs; n = 12 IPSPs, mean ± SEM, ANOVA). All comparisons: * ≙ p < 0.05, ** ≙ p < 0.01, *** ≙ p < 0.001 and **** ≙ p < 0.0001. Values are shown in S6 Data. (TIF) [file pbio.2001392.s006.tif]

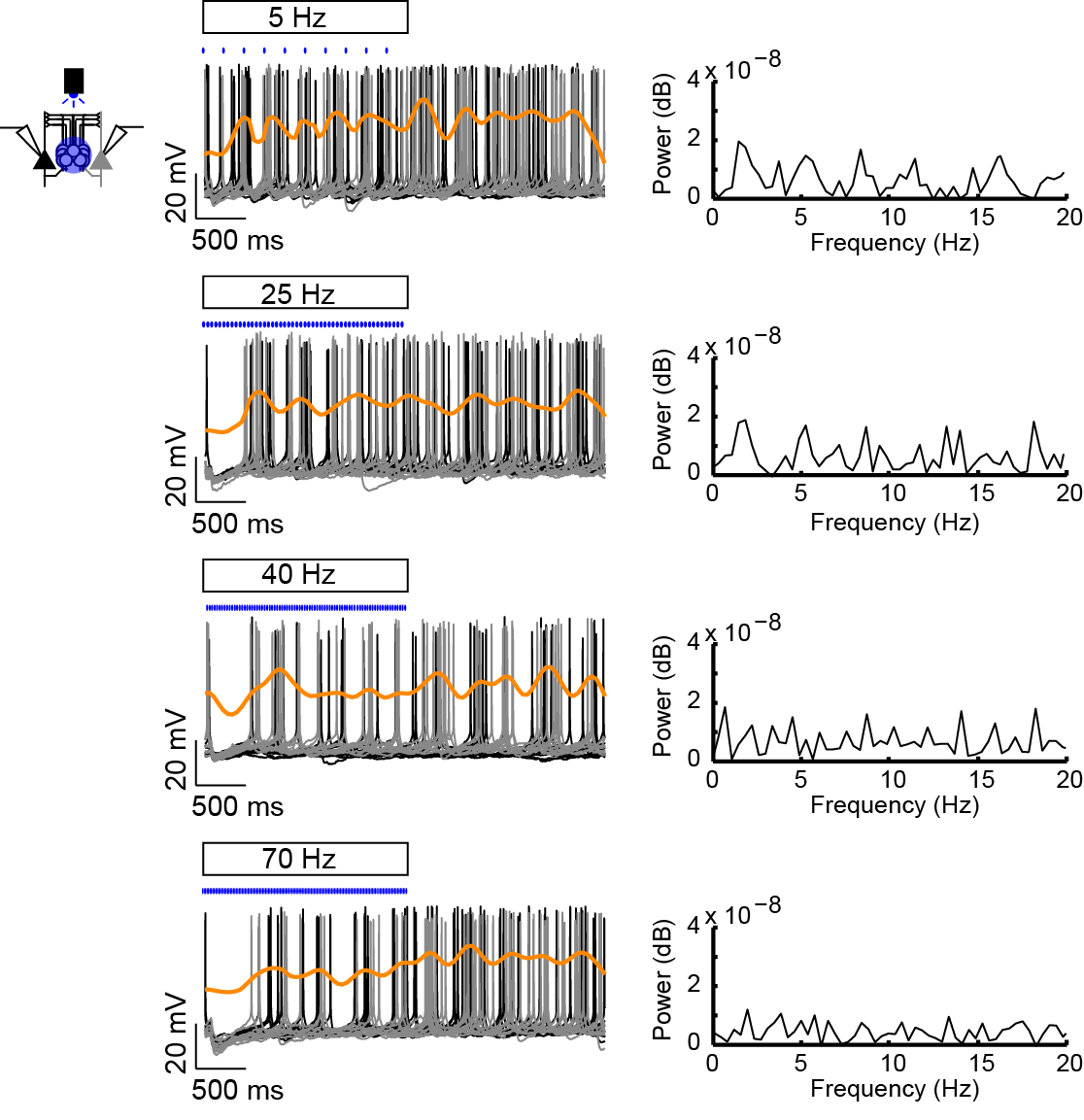

Supplement: S7 Fig — Population response of 12 dual recordings of unconnected type A PCs (left, n = 24 cells; 12 black and 12 grey PC spike trains) during different frequencies of pulsed light stimulation (5 Hz, 25 Hz, 40 Hz and 70 Hz) of ChR2-expressing MCsα2 and without light stimulation (second half of spike train), to compare if MC activity could synchronize PC firing. Kernel density estimates (orange trace) show increased (peaks) and decreased (valleys) co-occurances of APs. Mean power spectral density plots for each tested frequency (right) revealed no particular peak that indicated increased synchronization for 5, 25, 40 or 70 Hz. (TIF) [file pbio.2001392.s007.tif]

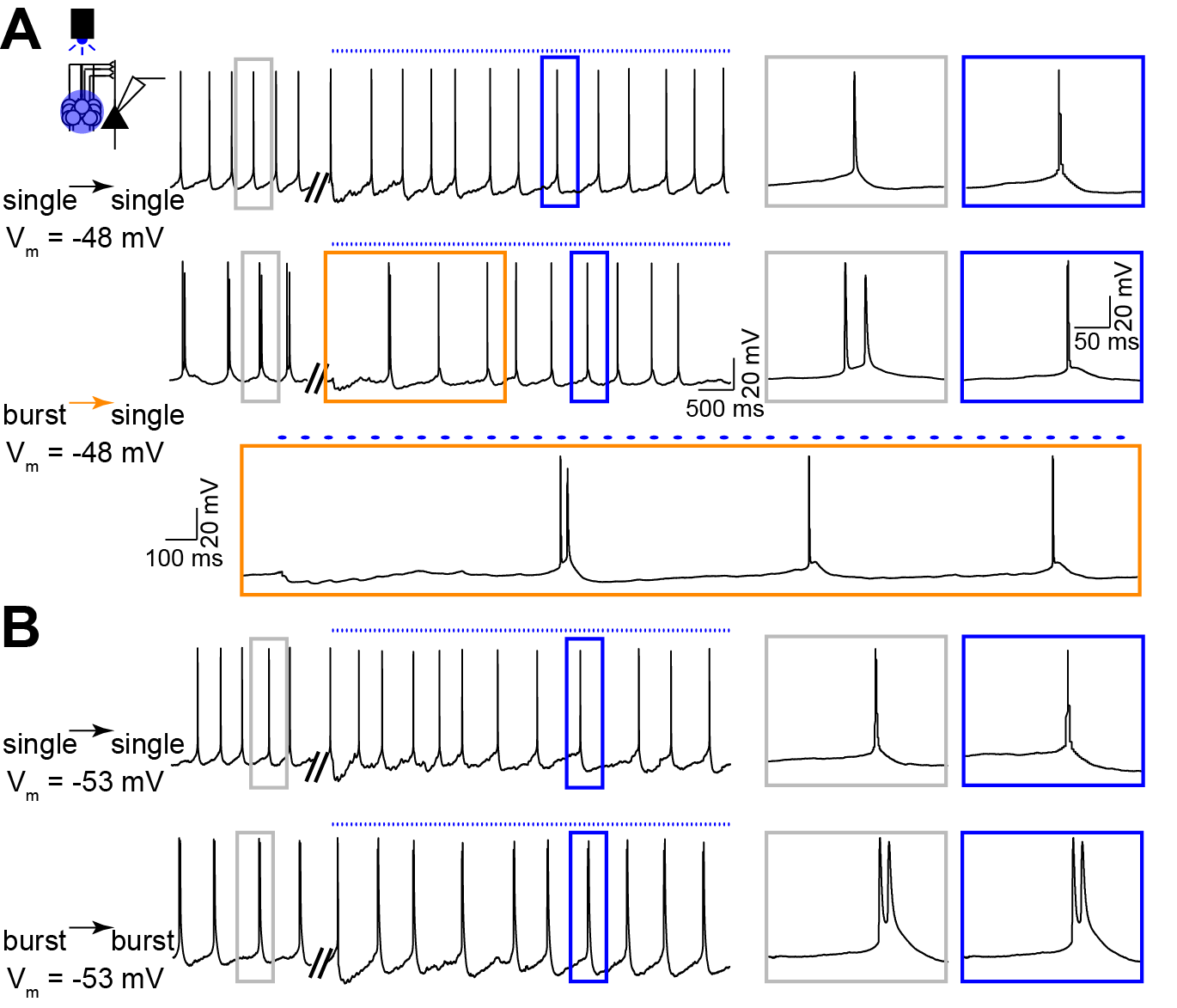

Supplement: S8 Fig — (A) Inset: Experimental set up and indication of how the PC firing pattern is altered/not altered by 15Hz MCsα2 activity. Voltage traces of a single-spiking (top) and a burst-spiking (bottom) type A PC at Vm = -48 mV (bath application of carbachol). Frames highlight typical APs. Bottom orange box; Note the change from doublet-spiking to single-spiking shortly after the initiation of MCsα2 inhibition at 15 Hz (by activating ChR2-expressing MCsα2 as indicated by blue dots above traces). (B) Voltage traces in (A) but at Vm = -53 mV shows that the change of firing pattern only happens at depolarized potentials, probably due to the stronger inhibition (larger Cl- drive at depolarized potentials). Frames highlight examples of APs at a zoomed in timescale. (TIF) [file pbio.2001392.s008.tif]
