# Supplementary material for: Chrna2-Martinotti Cells Synchronize Layer 5 Type A Pyramidal Cells via Rebound Excitation
Source: PLoS Biol. 2017 Feb 9;15(2):e2001392. doi: 10.1371/journal.pbio.2001392 (PMC5300109; doi:10.1371/journal.pbio.2001392)
Supplement: S1 Text — (DOCX) [file pbio.2001392.s019.docx]

**Supporting Information**

**Chrna2-Martinotti Cells Synchronize layer 5 type A**

**Pyramidal Cells via Rebound Excitation**Markus M. Hilscher, Richardson N. Leão, Steven J. Edwards, Katarina E. Leão, Klas Kullander

**S1 Fig. Tomato+ cells of Chrna2-Cre/*R26^tom^* mice across cortical areas.** (A) *Left*; 4x coronal images (60 µm thick) of primary auditory cortex and (and parts of secondary visual cortex – left image; *top*). Cell bodies of tdTomato+ neurons (red) appear in layer 5 and dense axonal arborizations are shown in layer 1 (image at approx. bregma -2.46 mm). The *star* highlights the oriens layer of the hippocampus and *arrowhead* shows dense axonal projections of oriens lacunosum-moleculare cells [19]. *Right*; 10x magnification of square area outlined in *left*. (B) Coronal slices (4x (*left*) and 10x (*right*) magnification) of the medial prefrontal cortex where the corpus callosum was not yet joined (around bregma +1.78 mm). (C) Parasagittal slices (10° angle), approximately 1.92 mm lateral to the midline (4x (*left*) and 10x (*right*) magnification), showing distribution of tdTomato+ cell bodies (red) in the primary somatosensory cortex, primary motor cortex and secondary visual cortex. Red squares show the approximate location of the 4x image (inset), white dashed squares for the 10x images. Note the dense axonal ramifications of Chrna2-Cre/*R26^tom^* cells in layer 1 (star). Scale bars = 400 μm (*left*) and 200 μm (*right*) resp. (D) Immunohistochemistry for somatostatin (left) in a cortical section from a Chrna2-Cre/*R26^tom^* mouse (middle, *star* highlighting the dense axonal ramifications of Chrna2-Cre/*R26^tom^* cells in layer 1) to visualize co-expression with chrna2 (right, arrowheads). Scale bar = 100 µm. (E) A total 792 cells were counted; 297 cells were Chrna2+, 495 were somatostatin+ and 90 of these were double labelled for both Chrna2 and somatostatin (n=3 mice, 8 sections of 35 µm thickness). Venn diagrams for all layers (layer1-6) and layer 5 visualize the overlap (somatostatin – grey; chrna2 – red and co-expression -pink). Insets in top corner of all panels show mouse brain atlas schematics of area show. (F) Electrophoresis gel image from the single cell analysis showing 6 positive cells (columns 2-4 and 6-8) for GAD1+, 1 negative cell (column 5) and the negative control (column 1).


**S2 Fig. Chrna2-Cre/*R26^tom^* cells show typical Martinotti cell morphology and putative interconnections with PCs.** (A) Example of biocytin-filled (green) Chrna2-Cre/*R26^tom^* cell highlighting the long axonal projection to layer 1 (*left,* 🡪) emerging from the main dendrite (*right*, circle). Note thick main trunks of dendrites of other red Chrna2-Cre/*R26^tom^* cell in the vicinity also pointing in the direction of layer 1. (B) Overview of the long axonal projection (🡪) of a biocytin filled (green) Chrna2-Cre/*R26^tom^* cell, showing proximal axonal arborizations (🡪) with main axons extending to layer 1. Note the dense axonal ramifications in layer 1 (star). (C) i) High magnification image (63x) of layer 1 (showing biocytin-filled (green) projections from one filled thick-tufted PC and a MC^α2^ cell, also green-yellow. The thin green-yellow MC^α2^ axon (highlighted with 🡪) could be followed visually and the high magnification image shows that it passes in close proximity to the thick dendrite of the PC, which was shown to be synaptically coupled with the recorded MC^α2^. The image is a collapsed z-stack composed of 40 (1 μm sections). ii) Close-up of the image in (i) but only showing collapsed z-stack of 10 images, to give a higher resolution, and still provide a pseudo 3D image of putative connections between the thin axon of the MC^α2^ and the thick dendrite of the PC. iii) Image showing the corresponding cell bodies of the PC and MC^α2^ (yellow) in the images on the left. Note also putative connections (arrow) from the PC to the red (not patched) chrna2-Cre/*R26^tom^* cell in the lower part of the image. Scale bars = 20 μm. **S3 Fig. MCs^α2^ are consistently activated by short duration blue light pulses and accommodating during continuous blue light stimulation.** (A). Comparison of evoked IPSPs in type A PCs following (*left*) action potentials generated by brief current injection (50 pA, 3 ms) in a connected MC^α2^, and (*right*) brief light stimulation (488 nm, 3 ms) of a population of ChR2+ MC^α2^. Graphs show comparison between amplitude, time to peak and half decay time of electrically (white) and optogenetically (blue) evoked IPSPs (n=12 cells, n=54 IPSPs with outliers removed, see methods). Values are shown in S2 Data. All comparisons: *** ≙ p < 0.001, mean ± SEM, two-tailed Student’s paired t-test. (B) Continuous blue light stimulation (500 ms) generates adaptation in MC^α2^ firing. (C) Continuous blue light of 1000 ms fails to generate prolonged firing in ChR2-expressing MCs^α2^. (D) Increasing light intensities (500 ms continuous blue light between 0.5 and 6 mW) does not improve spike capability of ChR2-expressing MCs^α2^. **S4 Fig. Properties of HaloR+ MCs^α2^ rebound spikes.** (A) Rebound APs of HaloR+ MCs^α2^ following different durations (100, 250, 500 and 1000 ms) of continuous green light (12 repetitions) in the presence of carbachol (V_m_ = -48 mV). Note that 100 ms of green light fails to consistently evoke rebound APs (failures highlighted by arrow), while 500 ms light was sufficient to generate a burst of rebound spikes. (B) Bar graphs show quantifications of number of rebound spikes, the peak hyperpolarization amplitude, rebound maximum frequency, rebound duration and time to peak of hyperpolarization. A 500 ms light stimulation was necessary to generate a burst of rebound spikes (>1), the hyperpolarization amplitude (during light) reached a plateau of -26.66 ± 0.17 mV (*top right*) (n=12 cells, mean ± SEM, ANOVA). 1000 ms light does not increase maximum frequency of rebound APs but increases the rebound duration (*bottom left*) (n=12 cells, mean ± SEM, two-tailed Student’s paired t-test). Quantification of hyperpolarization time to peak shows that there is no difference ≥ 250ms of light stimulation. All comparisons: * ≙ p < 0.05, ** ≙ p < 0.01, *** ≙ p < 0.001 and **** ≙ p < 0.0001. Values are shown in S5 Data. (C) MC^α2^ rebound APs generated by either negative current steps (0 – 100 pA) or by 500 ms green light are both resistant to ZD7288 (20 μM), a blocker of the hyperpolarization-activated cation current (I_h_).


**S5 Fig. Comparison of depolarization induced and carbachol induced firing of type A PCs and MCs^α2^.** Power spectral density plots (95% confidence interval in grey, mean in black) of type A PCs (*top*) and MCs^α2^ (*bottom*) are shown in response to a continuous (+40 pA) current injection (*left*) and following (*right*) carbachol (10 μM) bath application. Continuously adding carbachol to the perfusate increased the spontaneous firing frequency of both cells, with a broad peak (therefore not at any specific frequency) in the power spectrum at 4.90 Hz (range: 0.62 to 9.39 Hz, n=60 cells) for type A PCs and 16.02 Hz (range: 9.16 to 23.65 Hz, n=24 cells) for MCs^α2^.


**S6 Fig. Characteristics of compound IPSPs generated by a burst of APs of HaloR-expressing MCs^α2^.** (A) *Top*; Schematic of circuit. Green light stimulation (500 or 1000 ms) hyperpolarizes HaloR-expressing MCs^α2^ and upon termination of light the MCs^α2^ fired a burst of rebound APs. The corresponding compound IPSP in type A PCs represent the response to the first 3-4 MCs^α2^ spikes. Grey dashed lines highlight nicks in the trace where individual IPSPs are summed. (B) Example traces of type A PC IPSPs (grey traces, mean response in black) evoked by rebound APs following silencing a population of HaloR-expressing MCs^α2^ by green light of different duration (*left*: 500 ms; *right*: 1000 ms) for burst-spiking and (*top*) single-spiking (*bottom*) type A PCs. (C) IPSP amplitudes (*left*), time to peak (*middle*) and half decay time (*right*) in single-spiking and burst-spiking type A PCs vary depending on the light-duration (i.e. time of MC^α2^ inhibition and the subsequent rebound APs; n=12 IPSPs, mean ± SEM, ANOVA). All comparisons: * ≙ p < 0.05, ** ≙ p < 0.01, *** ≙ p < 0.001 and **** ≙ p < 0.0001. Values are shown in S6 Data.

**S7 Fig. Stimulation frequencies for MCs^α2^ that do not lead to synchronization of type A PCs.** Population response of 12 dual recordings of unconnected type A PCs (left, n=24 cells; 12 black and 12 grey PC spike trains) during different frequencies of pulsed light stimulation (5 Hz, 25 Hz, 40 Hz and 70 Hz) of ChR2-expressing MCs^α2^ and without light stimulation (second half of spike train), to compare if MC activity could synchronize PC firing. Kernel density estimates (orange trace) show increased (peaks) and decreased (valleys) co-occurances of APs. Mean power spectral density plots for each tested frequency (right) revealed no particular peak that indicated increased synchronization for 5, 25, 40 or 70 Hz.


**S8 Fig. Firing pattern of depolarized layer 5 PCs change from bursting to non-bursting upon inhibition by MCs^α2^ at 15 Hz.** (A) *Inset*: Experimental set up and indication of how the PC firing pattern is altered/not altered by 15Hz MCs^α2^ activity. Voltage traces of a single-spiking (*top*) and a burst-spiking (*bottom*) type A PC at V_m_ = -48 mV (bath application of carbachol). Frames highlight typical APs. *Bottom orange box*; Note the change from doublet-spiking to single-spiking shortly after the initiation of MCs^α2^ inhibition at 15 Hz (by activating ChR2-expressing MCs^α2^ as indicated by blue dots above traces). (B) Voltage traces in (A) but at V_m_ = -53 mV shows that the change of firing pattern only happens at depolarized potentials, probably due to the stronger inhibition (larger Cl^-^ drive at depolarized potentials). Frames highlight examples of APs at a zoomed in timescale.


**S1 Movie. Tomato+ cells of Chrna2-Cre/*R26^tom^* mice visualized across cortical areas.** A series of images from adult (2 months old) Chrna2-Cre/*R26^tom^* mouse cortex (coronal slice, 1300 µm thickness) after CLARITY processing is shown. Please note the second band of tomato+ cells highlighted in the stratum oriens of hippocampus [19] and the dense axonal arborization in stratum lacunosum-moleculare, highlighted as a grey dense mass.
